# The Contrasting Role of Marine‐ and Land‐Terminating Glaciers on Biogeochemical Cycles in Kongsfjorden, Svalbard

**DOI:** 10.1029/2023GB008087

**Published:** 2025-01-06

**Authors:** C. E. Schmidt, D. Pröfrock, G. Steinhoefel, T. Stichel, C. Mears, L. M. Wehrmann, H. Thomas

**Affiliations:** ^1^ Helmholtz‐Zentrum Hereon Institute of Carbon Cycles Geesthacht Germany; ^2^ Carl von Ossietzky University Oldenburg Institute for Chemistry and Biology of the Marine Environment Oldenburg Germany; ^3^ Helmholtz‐Zentrum Hereon Institute of Coastal Environmental Chemistry Geesthacht Germany; ^4^ Helmholtz Center for Polar and Marine Research Alfred Wegener Institute Bremerhaven Germany; ^5^ School of Marine and Atmospheric Sciences Stony Brook University Stony Brook NY USA

**Keywords:** Arctic Ocean, biogeochemistry, fjord, nutrients, carbon cycle, trace elements

## Abstract

This case study of Kongsfjorden, western coastal Svalbard, provides insights on how freshwater runoff from marine‐ and land‐terminating glaciers influences the biogeochemical cycles and distribution patterns of carbon, nutrients, and trace elements in an Arctic fjord system. We collected samples from the water column at stations along the fjord axis and proglacial river catchments, and analyzed concentrations of dissolved trace elements, together with dissolved nutrients, as well as alkalinity and dissolved inorganic carbon. Statistical tools were applied to identify and quantify biogeochemical processes within the fjord that govern the constituent distributions. Our results suggest that the glacier type affects nutrient availability and, therefore, primary production. Glacial discharge from both marine‐terminating glaciers and riverine discharge from land‐terminating glaciers are important sources of dissolved trace elements (dAl, dMn, dCo, dNi, dCu, and dPb) that are involved in biological and scavenging processes within marine systems. We identified benthic fluxes across the sediment‐water interface to supply fjord waters with silicate, dFe, dCu, and dZn. Our data show that intensive carbonate weathering in proglacial catchments supplies fjord waters with additional dissolved carbonates and, therefore, attenuates reduced buffering capacities caused by glacial runoff. Our study provides valuable insight into biogeochemical processes and carbon cycling within a climate‐sensitive, high‐latitude fjord region, which may help predict Arctic ecosystem changes in the future.

## Key Points

- Nutrient, trace element, and carbon species distributions are influenced by glacial discharge of land‐ and marine‐terminating glaciers
- Discharge of marine‐ and land‐terminating glaciers is an important source of bio‐essential trace elements
- Progressive glacier retreat will likely impact biotic and abiotic carbon uptake in the future

## 1 Introduction

In the Arctic Ocean, biogeochemical processes are heavily influenced by freshwater supply from atmospheric (water vapor, precipitation) and terrestrial sources (rivers, melting glaciers, and sea ice) (AMAP, 2017). Therefore, fjord systems act as an important link between the terrestrial domain and coastal waters on the adjacent shelf (Bianchi et al., 2020; Cottier et al., 2010). With increased terrestrial runoff predicted as a result of climate change (Carmack et al., 2016), it is important to consider how freshwater influx from various terrestrial sources affects biotic systems and natural habitats in coastal environments (Husum et al., 2019). It has been shown that the presence of either land‐ or marine‐terminating glaciers leads to differences in the ecosystem productivity of high‐latitude fjord regions (Hopwood et al., 2020; Meire et al., 2017). Specifically, subglacial discharge originating from beneath marine‐terminating glaciers causes the entrainment and subsequent upwelling of large volumes of nutrient‐rich deep water (Halbach et al., 2019; Hopwood et al., 2018; Meire et al., 2017; Meslard et al., 2018). This vertical flux supplies macronutrients (nitrate, NO_3_^−^; nitrite, NO_2_^−^; phosphate, PO_4_^3−^; silicate, Si(OH)_4_) to shelf and slope waters and leads to an overall higher productivity in these areas (Cape et al., 2019; Hawkings et al., 2015; Hendry et al., 2019; Meslard et al., 2018; Trusel et al., 2010). In contrast, fjords with land‐terminating glaciers show lower productivity as they lack this entrainment mechanism (Meire et al., 2017). Without upwelling and entrainment, freshwater runoff from proglacial rivers enhances stratification, which restricts nutrient mixing from deeper waters, resulting in low macronutrient availability throughout summer (Hopwood et al., 2020; Meire et al., 2017; Santos‐Garcia et al., 2022). However, intense chemical and physical weathering in proglacial catchments can also result in a high flux of macro‐ and micronutrients and other dissolved elements in riverine runoff, which can be beneficial for the biogeochemical functioning of fjords (Rutter et al., 2011).

Many dissolved trace elements are essential micronutrients (dMn, dFe, dCo, dNi, dCu, dZn, dCd) that play critical roles in biological processes, for example, the photosynthetic fixation of inorganic carbon (Emerson & Hamme, 2022; Morel et al., 2014; Smrzka et al., 2019). As such, it is important to study sources and sinks of bio‐active elements, especially in rapidly changing environments such as Arctic coastal regions (Gerringa et al., 2021). Terrestrial freshwater discharge is an important source of trace elements in fjord environments (Hawkings et al., 2020; Krause et al., 2021). The elemental concentration of freshwater depends not only on the local bedrock geology (Krause et al., 2021), but also on the length and intensity of the melt season that drives water‐rock interactions (Aciego et al., 2015). Overall, the distribution and bioavailability of freshwater derived trace elements depend on their behavior during estuarine mixing (Hawkings et al., 2020). There are two pathways by which trace elements are converted from the dissolved (d; <0.22 μm) to the particulate phase: (a) active uptake during biological cycling and (b) passive scavenging onto particle surfaces (Bruland et al., 2014; Hawkings et al., 2020; Krause et al., 2021). For example, the distribution of the trace element Fe in coastal zones of the Arctic has been shown to be influenced by different physicochemical processes in a number of studies (Kanna et al., 2020; Markussen et al., 2016; Schroth et al., 2014). Similar to macronutrients outlined above, subglacial discharge drives the upwelling of dFe toward the euphotic zone, where it can be used as an essential micronutrient for primary production (Kanna et al., 2020). An equally important mechanism for the distribution of Fe in high‐latitude fjord systems is the extensive estuarine removal of glacial derived dFe (Schroth et al., 2014), which creates labile Fe particles that can be transported horizontally and exported to marine environments (Markussen et al., 2016).

Spatio‐temporal variations of terrestrial freshwater sources greatly influence the ocean chemistry of high latitude fjord and coastal environments, particularly regarding the effects of ocean acidification (OA) (Jones et al., 2020). Because of low carbonate ion concentrations, meltwater discharge generally reduces alkalinity (AT) and dissolved inorganic carbon (CT) concentrations through dilution, thus reducing the buffering capacity of fjord waters (Chierici & Fransson, 2009; Fransson et al., 2015; Hopwood et al., 2020; Shadwick et al., 2011). This makes high latitude surface waters particularly sensitive to increases in atmospheric CO_2_ uptake (Chierici & Fransson, 2009) and enhances surface water acidification in coastal and seasonally ice‐covered regions (Chierici & Fransson, 2009; Jones et al., 2020; Reisdorph & Mathis, 2014). Reduced carbonate mineral saturation states (Ω) were found to correlate with the timing of maximum glacial discharge (Reisdorph & Mathis, 2014) and the amount of freshwater discharge (Fransson et al., 2015), and were most prominent in regions with marine‐terminating glaciers (Reisdorph & Mathis, 2014). The corresponding reduction in Ω causes physiological stress for calcifying organisms, as these waters become more corrosive, which leads to dissolution and thinning of shells (Fransson et al., 2016; Reisdorph & Mathis, 2014). However, freshwater from proglacial catchments characterized by bedrock abundant in carbonate and silicate minerals has the potential to partly mitigate OA and lower Ω values to some extent (Fransson et al., 2015).

Svalbard is a highly glaciated archipelago on the northwestern Eurasian continental shelf between 74 and 81°N (Dallmann, 2015). In this study, we chose Kongsfjorden (Western Svalbard; 79°N, 11–13°E) as a study site, which is known to be a well‐monitored natural laboratory for Arctic marine studies (Wiencke & Hop, 2016). The Kongsfjorden system already experiences strong impacts of larger climate‐driven processes and thus can be considered a high‐latitude reference site for future changes (Bischof et al., 2019). The fjord is surrounded by land‐ and marine‐terminating glaciers and is largely influenced by terrestrial freshwater supply (Svendsen et al., 2002) as well as the inflow of warm and saline Atlantic waters (AW) (Luckman et al., 2015; Promińska et al., 2017), making Kongsfjorden a suitable study area to investigate biogeochemical processes within glacier‐dominated fjord systems.

Previous studies investigated trace elements in surface sediments of Kongsfjorden with a focus on the particulate fraction (e.g., Ardini et al., 2016; Bazzano et al., 2014; Grotti et al., 2013; Herbert et al., 2021; Wehrmann et al., 2014; Zaborska et al., 2017). Wehrmann et al. (2014) found that the composition of the catchment bedrock influences the input of dFe and Fe(III) and Mn(III/IV) oxide‐hydroxide phases to the fjord. Mechanical and biogeochemical processing of Fe‐ and pyrite‐rich rock types, such as sandstone and shale, produces high‐iron glacial flour and results in high Fe delivery to sediments (Wehrmann et al., 2014). Studies that focus on the distribution of dissolved trace elements in the water column of Kongsfjorden are very limited. Glacial meltwater has been found to serve as a primary source of dAl, dFe (Shen et al., 2024), and dMn (Yang et al., 2022) to Kongsfjorden. The distribution of dAl (Shen et al., 2024) and dMn (Yang et al., 2022) is dominated by conservative mixing, while the distribution of dFe is influenced by more complex processes within the fjord, such as particle interactions and biological activity (Shen et al., 2024). Proglacial rivers have been identified as a minor source of dFe (Zhang et al., 2015) and dMn (Yang et al., 2022) to Kongsfjorden, but pose an important source of reactive particulate phases that can be reduced in marine sediments (Herbert et al., 2022; Zhang et al., 2015). To the best of our knowledge, the distributions of other dissolved trace elements with importance for biogeochemical processes have not been studied in Kongsfjorden yet.

Concerning the carbonate system in Kongsfjorden, Fransson et al. (2016) showed that the inflow of different water masses and terrestrial freshwater directly affects the carbonate system and indirectly affects the biological uptake of CO_2_. Overall, glacier discharge was found to be a strong local amplifier of OA, leading to corrosive conditions at low salinity in the inner fjord during summer (Cantoni et al., 2020). In contrast, as weathering in the catchment is dominated by carbonate dissolution in combination with limited silicate weathering (Hindshaw et al., 2016; Krawczyk et al., 2003; Kumar et al., 2018; Stutter & Billett, 2003), freshwater from proglacial rivers could counteract the dilution of AT and CT, and the effects of OA by enhanced weathering in the catchment (Chierici & Fransson, 2009; Koziorowska‐Makuch et al., 2023). This has the potential to provide a partly negative feedback to OA, while increasing the capacity for atmospheric CO_2_ uptake (Fransson et al., 2015). Previous studies point out the complexity of the carbonate system, advertising the need for more data, especially with regard to freshwater sampling and endmember characteristics.

Given the above considerations, the aim of this study is to understand the linkage between terrestrial runoff from different freshwater sources and the biogeochemical processes that affect nutrient, carbon, and trace element distributions in the water column of high‐latitude fjords. We hypothesize that there is a distinction between the chemical effects of the marine‐ and land‐terminating glaciers in terms of endmember characteristics and between the physical effects of the different glacier systems with regard to cycling and redistribution of dissolved constituents, which potentially has larger consequences for primary production. We use multivariate statistical methods, namely principal component analysis (PCA), cluster analysis and absolute principal component score‐multiple linear regression (APCS‐MLR), to identify and quantify biogeochemical processes that change fjord distributions of dissolved elements and nutrients. Our objectives are to: (a) characterize the spatial distribution of inorganic carbon, dissolved trace elements, and nutrients in fjord waters; (b) identify; and (c) quantify how biogeochemical processes influence these distributions in Kongsfjorden.

## 2 Materials and Methods

### 2.1 Study Area

**Figure 1:**
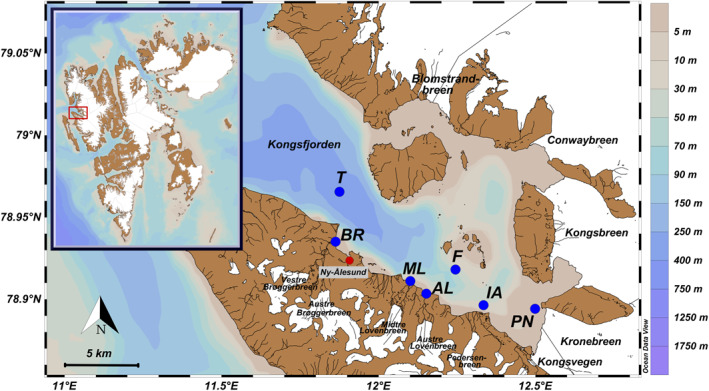
Map of Kongsfjorden with blue points indicating the location of water column sampling stations (T, F, IA, and PN) and of freshwater runoff sampling locations from proglacial rivers (BR, ML, and AL).

The investigated fjord Kongsfjorden is located on the west coast of the Svalbard archipelago (79°N, 11–13°E). The fjord is surrounded by land‐ and marine‐terminating glaciers and opens toward the Greenland Sea in the west (Svendsen et al., 2002). The hydrography of Kongsfjorden is strongly influenced by terrestrial freshwater supply (Svendsen et al., 2002) as well as the inflow of warm and saline Atlantic waters (AW) of the West‐Spitsbergen Current (WSC) (Luckman et al., 2015; Promińska et al., 2017). Bottom waters of the fjord are affected by the intrusion of AW from the WSC (De Rovere et al., 2022). In the time period 2010–2020, De Rovere et al. (2022) observed an Atlantification of Kongsfjorden due to enhanced AW advection. This becomes apparent with increasing heat and salt content of the water column, which potentially triggers amplified ablation rates of marine‐terminating glaciers draining into Kongsfjorden and thus increases freshwater content (De Rovere et al., 2022; Luckman et al., 2015). During the melt season (June–August), freshwater input from marine‐ and land‐terminating glaciers, snowmelt and summer precipitation affect the fjord surface water masses, creating a stable stratification that influences the physical, chemical and biological processes (David & Krishnan, 2017; Svendsen et al., 2002). The estimated average volume of freshwater supply to Kongsfjorden ranges between 0.03 km^3^ in 2014 and 0.42 km^3^ in 2011, which represents between 0.12% and 1.5% of the fjord’s volume (Promińska et al., 2017). There are four marine‐terminating glaciers in the inner part of Kongsfjorden: two located at the fjords head, Kronebreen‐Kongsvegen and Kongsbreen, and two situated along its northern coast, Conwaybreen and Blomstrandbreen (Figure 1). Furthermore, the southern region of Kongsfjorden is characterized by land‐terminating glaciers that drain through proglacial valleys into the fjord (Pramanik et al., 2018). Kronebreen is by far the fastest flowing glacier on Svalbard with a velocity of 700–800 m per year (Błaszczyk et al., 2009; Schellenberger et al., 2015), contributing around 39% of the total freshwater to Kongsfjorden (Pramanik et al., 2020). Discharge at the Kronebreen outlet usually starts in early June, continues through July and August, and ends in early September (Pramanik et al., 2020). In contrast to the marine‐terminating glaciers, annual runoff from the land‐terminating glaciers is about one order of magnitude smaller (Pramanik et al., 2020). The Bayelva catchment, which is fed by the most westerly land‐terminating glacier, Brøggerbreen, accounts for the largest amount of freshwater from the land‐terminating glaciers (Pramanik et al., 2020). Discharge at the Bayelva river starts slightly earlier than at Kronebreen at the end of May and continues until late September (Pramanik et al., 2020). Other land‐terminating glaciers that recharge proglacial rivers are the Lovénbreen system and the Pedersenbreen glacier (Figure 1). In the time period 2002–2021, a long‐term mass loss of 13.2 ± 2.3 Gt · yr^−1^ was observed for the entire Svalbard area due to the intensification of meridional heat advection (Sasgen et al., 2022). The overall accelerated glacier mass loss is promoted by the significant increase in air and ocean temperatures (Wang et al., 2022), with simulated runoff rates from glaciers being largest in 2013 and 2020 (Schmidt et al., 2023). In Kongsfjorden, historical glacier mass balance measurements of Brøggerbreen and Lovénbreen show a predominantly negative mass balance in the over 40‐year long record (Dallmann, 2015). The seasonal mean temperature at Svalbard Airport for summer (June‐July‐August) in 2020 was relatively high at 7.2°C (Norwegian Meteorological Institute, 2022), resulting in high freshwater influxes as seen by an extreme loss of the annual mass balance for Brøggerbreen and Lovénbreen in 2019/2020 (WGMS, 2024).

### 2.2 Sampling

Fjord water and freshwater samples were collected from Kongsfjorden and the area surrounding Ny‐Ålesund during six consecutive sampling days in early July 2020 (Figure 1, Table S1 in Supporting Information S1) during the middle of the melting season. We sampled the water column along the fjord axis based on relative distance to the marine‐terminating Kronebreen‐Kongsvegen system to cover locations at the inner fjord within close proximity to the main upwelling plume of Kronebreen (station PN), mid fjord (station F), and outer fjord (station T), as well as in proximity to the proglacial runoff from the land‐terminating Pedersenbreen glacier (station IA). In total, 14 fjord water samples at three to four different depths were collected on board the AWIPEV research vessel Jean Floch using a 12 L trace‐metal clean Niskin water sampler (General Oceanics; Florida, USA) attached to a CTD profiler (Model SD204, SAIV A/S; Laksevag, Norway) using 3 m of Nylon rope. Before sampling in Kongsfjorden, the Niskin bottle was treated by several cleaning steps involving type I reagent‐grade water, alkaline detergent, diluted HNO_3_ and HCl. In addition, we sampled the main meltwater rivers of three catchments with land‐terminating glaciers: Bayelva River (BR), Midtre Lovénbreen (ML) and Austre Lovénbreen (AL). Detailed information about each sample is given in Table S1 of the Supporting Information S1 with sample IDs corresponding to the respective station and sampling depth. Field measurements included recording temperature, salinity and pH with a multi‐parameter portable meter (Multi 340i IDS, WTW; Weilheim, Germany). For pH, the sensor was calibrated before each field day under laboratory conditions at 20°C, using calibration solutions at pH 7 and 10 (technical buffer solution, WTW; Weilheim, Germany). Bulk samples for nutrient and multi‐element analysis were filled into pre‐cleaned 2 L HDPE (high‐density polyethylene) bottles and further processed immediately after returning to the laboratory in Ny‐Ålesund before shipping to Germany. The time between sampling and filtration was less than 12 hr. Samples for AT and CT were collected in 300 mL BOD (biological oxygen demand) bottles with an addition of 300 μL saturated mercury chloride solution. The bottles were sealed with ground‐glass stoppers, Apiezon type M grease, and plastic caps, leaving no head space. The samples were stored in darkness at ambient temperature in the laboratory until analysis.

### 2.3 Sample Preparation and Chemical Analysis

All equipment for trace element sampling was acid washed prior to use and rinsed with type I reagent‐grade water (resistivity: 18.2 MΩ · cm) obtained from a Milli‐Q Integral water purification system (Merck; Darmstadt, Germany) to minimize contamination. Before transportation, samples for nutrient and multi‐element analysis were filtered in a class 100 clean bench through PVDF membrane filters (polyvinylidene difluoride membrane, 47 mm diameter × 0.22 μm pore size; Merck; Darmstadt, Germany) and collected in pre‐cleaned 250 and 1,000 mL HDPE bottles, respectively. Samples for multi‐element analysis were stabilized using trace metal grade HCl (Fisher Scientific; Waltham, USA) and stored in darkness at 4°C until analysis. Filtered nutrient samples were frozen until analysis. Nitrate, nitrite, silicate and phosphate in water samples were analyzed using a QuAAtro39 AutoAnalyzer (SEAL Analytica; Norderstedt, Germany) at the nutrient facility of the Alfred‐Wegener‐Institute in Bremerhaven. The reference material for nutrients in seawater (RMNS, Lot BZ and Lot CL) (Kanso Technos; Osaka, Japan) was used for method validation, and recovery rates are given in Table S3 of the Supporting Information S1. As eluent, 36 g · L^−1^ sodium chloride solution was prepared for seawater analysis and type I reagent‐grade water for freshwater samples. Limits of detection (LODs) were provided by the manufacturer and are given in Table of the Supporting Information S1. For nutrient analysis, water samples were measured as duplicates, and results are presented with uncertainties corresponding to a coverage factor *k* = 2. Dissolved trace elements (dAl, dV, dFe, dMn, dCo, dNi, dCu, dZn, dCd, and dPb) in seawater were measured at the Helmholtz‐Zentrum Hereon in Geesthacht by using a seaFAST SP2 system (Elemental Scientific; Omaha, USA) coupled online to an inductively coupled plasma mass spectrometer (ICP‐MS) system (Agilent 7900, Agilent Technologies; Tokyo, Japan) to eliminate the seawater matrix and corresponding interferences, as well as preconcentrate the target analytes which are present at low concentrations. The system contains two columns filled with Nobias chelate‐PA1 resin (HITACHI High‐Tech Fielding Corporation; Tokyo, Japan) for matrix removal and analyte enrichment with 4 mol · L^−1^ ammonia acetate buffer (pH = 6.0 ± 0.2) and 1.5 mol · L^−1^ HNO_3_ needed for the column chemistry, as well as eluent for preconcentrated analytes. The ICP‐MS instrument was optimized daily using a tuning solution containing Li, Co, Y, Ce, and Tl to maintain a reliable day‐to‐day performance. The system was operated in He/H_2_ mixed gas mode and equipped with an x‐lense to further minimize spectral interferences on the targeted analytes. Detailed operating parameters and instrument configurations are given in Table S2 of the Supporting Information S1. More information about the analytical procedure can be found in Ebeling et al. (2022). For method validation, the certified reference materials (CRMs) CASS‐6 for near shore seawater, NASS‐7 for open ocean seawater, and SLRS‐6 for river water (all provided by the National Research Council; Ottawa, Canada) were used. Additionally, an in‐house reference material (KBA‐QC), mixed from single element standards (Carl Roth GmbH, Karlsruhe, Germany or Sigma‐Aldrich, Missouri, USA) and custom‐made multi‐element standards (all traceable to NIST standards) of different compositions (Inorganic Ventures, Christiansburg, USA), was used to validate elements that are not covered by CRMs (e.g., dAl). Recovery rates (between 89% and 128%) are given in Table S3 of the Supporting Information S1. For multi‐element analysis, water samples were measured in triplicates. From there, combined uncertainties were calculated, considering the standard deviation of the triplicate and the precision of the measurement. Finally, uncertainties are given as expanded uncertainty with a coverage factor *k* = 2. Limits of detection (LOD) and limits of quantification (LOQ) were calculated according to DIN 32645:2008‐11 based on three method blanks, with LOD defined as 3x standard deviation (SD) and LOQ as 10x SD of the blank (DIN e.V., 2008). Measurements of AT and CT were performed using a VINDTA 3 C system (Marianda; Kiel, Germany) at the Helmholtz‐Zentrum Hereon in Geesthacht. Hereby, AT and CT are determined simultaneously by potentiometric titration using an 800 Dosino (Metrohm; Filderstadt, Germany) with an Aquatrode plus (Metrohm; Filderstadt, Germany) and coulometric detection using a CM5017O coulometer (UIC; Guilhabreu, Portugal), respectively. Both instruments were calibrated against seawater CRMs (Scripps Institution of Oceanography; San Diego, USA) to ensure a precision of ±1 μmol · kg^−1^ for AT and CT, respectively.

### 2.4 Data Analysis

Multi‐elemental data were pre‐processed using MassHunter version 4.6 (Agilent Technologies; Tokyo, Japan). Data pre‐treatment was executed with in‐house evaluation templates based on MS Excel 2016 (Microsoft Corporation). Parameters of the carbonate system (pH, pCO_2_, and Ω) were calculated using the CO2Sys Macro (Pierrot et al., 2011) for MS Excel 2016 (Microsoft Corporation) with salinity, temperature, AT and CT as input variables. As input parameters, we used the dissociation constants by Mehrbach et al. (1973), refit by Dickson and Millero (1987), the HSO_4_^−^ dissociation constants by Dickson (1990) and the seawater pH‐scale. Values below LOD were replaced with a random value between zero and LOD due to the limited availability of accurate measurements of those values. The data were standardized, and multivariate statistical analysis was performed using R (version 4.2.2) and RStudio (version 2022.12.0) with basic packages as well as tidyverse, dplyr, psych, grid and dendextend. As a first step, PCA with varimax rotation was applied to calculate PC scores and loadings to identify processes and trends that drive fjord distributions. The measured parameters were tested for normality (Table S4 in Supporting Information S1). PCA assumes that each variable is normally distributed, thus guaranteeing that the resulting components, in addition to being orthogonal, are independent. Multivariate normality of the variables is needed to guarantee the independence of principal components; that is, each component can be uniquely interpreted without being influenced by other components. The small sample size of this data set did not allow for the normalization assumption of PCA to be met for some parameters (salinity, AT, Al, V, Mn, Co, Ni, Pb). To account for this, we tested the independence of PCs (Table S5 in Supporting Information S1). Following the approach by Kim and Kim (2012), each component was discretized into four equal interval groups; then, we constructed 3 × 4 cross tables and ran the independence test. The test justified the independence of PCs; thus, we can assume that each component represents an exclusive trait. A number of three principal components (PCs) were selected, taking into account the Guttman‐Kaiser criterion and the trend of the scree plot. A broken stick analysis was performed to distinguish the loading significance of each variable. Afterward, PC loadings were used in cluster analysis using Ward's algorithm and squared Euclidean distances. Finally, results of the PCA were used to quantify the contribution of identified processes on fjord distributions in the study area using the approach of Thurston and Spengler (1985). Although the APCS‐MLR technique was originally developed to quantify major pollution sources for aerosols and particulate matter, more recently it has been used in water quality research to identify pollution sources from natural processes and anthropogenic activities in rivers of East China (K. Chen et al., 2022) and groundwater of the Yangtze River Delta (Z. Chen et al., 2023). A detailed summary of the statistical method is given in Appendix A. For the APCS‐MLR model, we chose the following significance codes to determine the statistically significant predictive capability of each factor: “***”: *P* < 0.001; “**”: *P* < 0.01; “*”: *P* < 0.05; and “·”: *P* < 0.1 with a cutoff value of *P* ≥ 0.1. If the cutoff value was reached, the factor was removed as a variable from the model and re‐run with the remaining variables. The significance codes do not indicate how large of an effect each factor has on the parameter but rather indicate how certain we can be that the factor has an impact on the dependent parameter. For example, a significance level of *P* < 0.1, indicates that there is less than a 10% chance that the coefficient might be equal to 0 and thus be insignificant. We chose a cutoff value of 10% since the number of observations is not very high, which poses the risk of overlooking smaller changes and losing valuable information if *P* is chosen too small. Freshwater calculations are adapted from the methodology described in Beszczynska‐Möller et al. (1997) and Promińska et al. (2017) and are shown in Appendix B. The freshwater content (FWC (m)) was calculated as the depth‐integrated difference between the measured salinity and the reference salinity (34.86). From this, the specific freshwater content (FWCsp (%)) was calculated as the proportion of freshwater per station by averaging FWC by depth. The map and cross sections of Kongsfjorden were drawn with Ocean Data View (version 5.6.2; (Schlitzer, 2022)) using shapefiles provided by the Norwegian Polar Institute (Norwegian Polar Institute, 2014). Linear and non‐linear regressions as well as definite integrals were calculated in Origin 2020b (OriginLab Corporation) with direct weighting of errors.

## 3 Results and Discussion

In the following sections, we will give a qualitative overview of observed distributions in Kongsfjorden, starting with water masses (Section 3.1) and continuing with macronutrients, parameters of the carbonate system, and trace elements (Section 3.2). We will proceed with the discussion by using statistical analysis to identify and quantify physicochemical processes that alter the distribution of carbon, nutrients, and trace elements (Section 3.3). In the final section, we will present endmember concentrations based on observed concentrations and those calculated from the statistical analysis (Section 3.4).

### 3.1 Water Mass Distribution in Kongsfjorden

The hydrographic data showed spatial variability in the water masses of Kongsfjorden. Water masses were classified according to Cottier et al. (2005) and are summarized in Table S6 of the Supporting Information S1.

**Figure 2:**
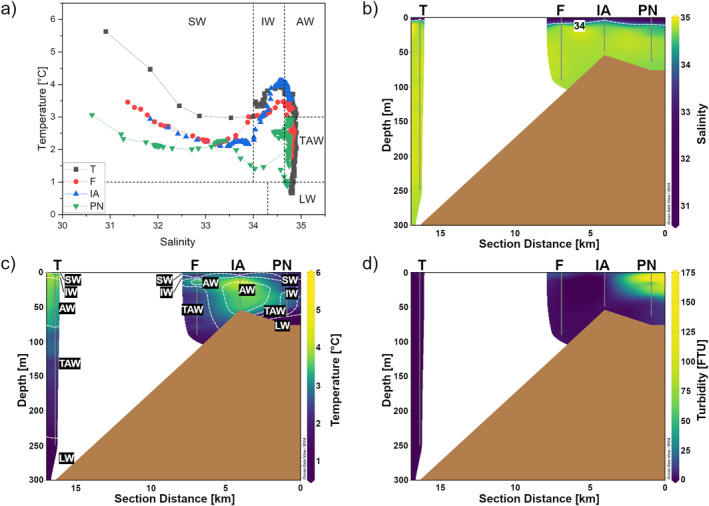
(a) T‐S diagram used to identify water masses of Kongsfjorden. Distinct water masses are indicated as Surface Water (SW), Intermediate Water (IW), Local Water (LW), Transformed Atlantic Water (TAW), and Atlantic Water (AW) as classified by Cottier et al. (2005). Station names are given as T, F, IA, and PN. (b) Vertical distribution of salinity with isohalines for 34; (c) vertical distribution of temperature overlain by water mass contours; and (d) vertical distribution of turbidity. The section distance refers to the approximate distance from the Kronebreen‐Kongsbreen terminus along the fjord axis.

Water masses in Kongsfjorden were characterized by different salinity and temperature properties (Figure 2a). Recent studies confirm the extensive intrusion of AW into the fjord during summer, causing the fjord to undergo a transition from Arctic‐ to more Atlantic‐type fjord (i.e., higher temperature and salinity) (De Rovere et al., 2022; Promińska et al., 2017). This was seen here, where the intrusion of AW along with local processes such as warming, glacial melt, mixing and entrainment resulted in the presence of four additional water masses: Transformed Atlantic Water (TAW), Surface Water (SW), Intermediate Water (IW), and Local Water (LW) (Cottier et al., 2005; David & Krishnan, 2017). As depicted in Figures 2b and 2c, AW occupied the water column between 10 and 80 m with a thin layer of IW separating it from SW by a strong halocline. The intrusion of warmer AW at intermediate depths coupled with colder and fresher SW originating from terrestrial freshwater runoff resulted in a temperature inversion of the water column (Figure 2c). Beneath the Kronebreen‐Kongsbreen system, Meslard et al. (2018) found evidence for an outflow of fresh and turbid meltwater originating from a large subglacial river. As the subglacial meltwater has a lower density than the surrounding waters, it was found to rise from the grounding line of the glacier at 60 m depth to the surface, creating a large turbid plume (Darlington, 2015; Meslard et al., 2018). This is illustrated in Figure 2d by means of high turbidity values between 0 and 50 m at station PN. Below the AW layer, TAW extended toward the seafloor with cold layers of high salinity waters of LW at the bottom. The much colder LW is formed during autumn and winter and can be found throughout the year at the bottom of deep basins and depressions (Cottier et al., 2005), which was confirmed by our results (Figures 2a and 2c). By the mixing of AW with LW, TAW is formed, which replaces LW in the fjord, as seen for stations F and T (Figure 2c).

The freshwater content (FWC) and the specific freshwater content (FWC_sp_) for each station were calculated. The values for FWC and FWC_sp_ are given in Supporting Information S1 (refer to Table S7 in Supporting Information S1). Representation of SW generally decreased toward the fjord mouth as the FWC_sp_ of each station decreased from 1.5% at station PN to 0.15% at station T (Table S7 in Supporting Information S1). When comparing the height of the freshwater column (FWC) between the stations, it becomes apparent that IA (0.43 m) received less freshwater than the other inner stations PN (0.90 m) and F (0.68 m) (Table S7 in Supporting Information S1). The smaller height of the freshwater column at IA can be explained by the different mean annual runoff rates to the Kongsfjorden basins, which are about 20 times less for the Lovén‐ and Pedersenbreen catchments than for Kronebreen and Kongsvegen (Svendsen et al., 2002). This coincides well with results by Torsvik et al. (2019), who found a larger volume of low‐salinity water on the northern side of the Kronebreen‐Kongsbreen system than on the southern side. As fresher surface waters flow out of the fjord, there is a compensatory influx of AW driven by local winds and estuarine circulation (Husum et al., 2019; Svendsen et al., 2002). The circulation model of Kongsfjorden by Torsvik et al. (2019) showed a strong outward flow toward the north‐west in front of the Kronebreen‐Kongsbreen system in surface waters (0–10 m). In the 20–30 m layer, the currents reversed with a strong inward flow toward the south‐east (Torsvik et al., 2019). Consequently, we suggest that the southern coast of Kongsfjorden was less influenced by the meltwater plume of the Kronebreen‐Kongsbreen system and more dominated by intrusion of AW and drainage from land‐terminating glaciers, as reflected in our data at station IA. The reduced influence of glacial runoff from Kronebreen‐Kongsbreen is also supported by a lower turbidity and specific freshwater content in surface waters of station IA compared to adjacent stations in the inner fjord (Figure 2d, Table S7 in Supporting Information S1).

### 3.2 Distribution of Carbon, Nutrients, and Trace Elements in Kongsfjorden

**Figure 3:**
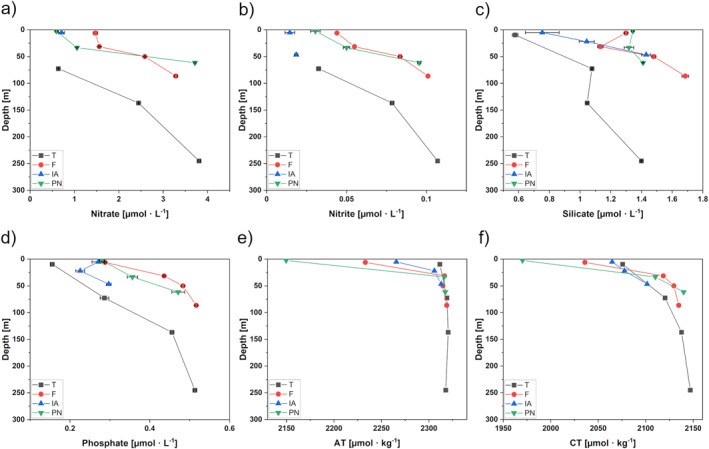
Depth profiles of selected parameters in Kongsfjorden for stations T (black square), F (red circle), IA (blue triangle), and PN (green triangle). The parameters shown are (a) nitrate, (b) nitrite, (c) silicate, (d) phosphate, (e) alkalinity (AT), and (f) dissolved inorganic carbon (CT). Values below LOD are not shown. Error bars correspond to U (*k* = 2). Error bars are not applicable to AT and CT results.

**Figure 4:**
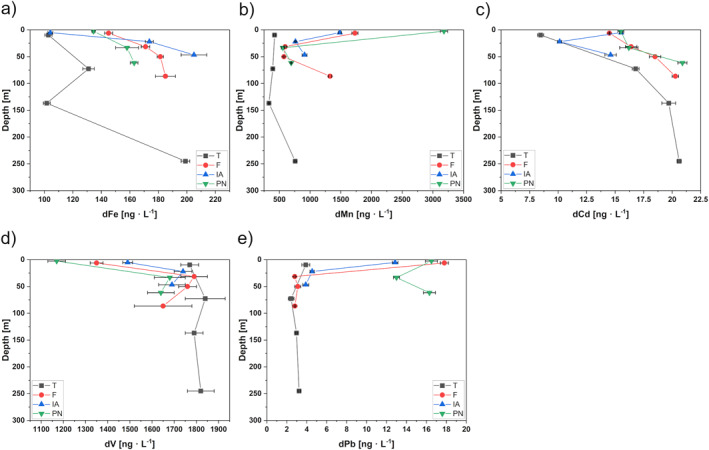
Depth profiles of selected trace elements in Kongsfjorden for stations T (black squares), F (red circles), IA (blue triangle), and PN (green triangle). Shown are dissolved trace elements: (a) Iron (dFe), (b) Manganese (dMn), (c) Cadmium (dCd), (d) Vanadium (dV), and (e) Lead (dPb). Values below LOQ are not shown. Error bars correspond to U (*k* = 2).

A compilation showing the depth profiles of measured nutrients, AT and CT in Kongsfjorden is given in Figure 3. We selected certain dissolved elements (dFe, dMn, dCd, dV, and dPb) for a detailed discussion based on their observed mixing behavior and availability of existing literature on their behavior in the water column of coastal systems (Figure 4). The remaining depth profiles are given in Figure S1 of the Supporting Information S1. Overall, we found similarities between the depth profiles of (a) dFe with dZn and dCu; and (b) dMn with dAl, dCo, and dNi. An overview of minimum and maximum concentrations over all fjord stations as well as concentrations in the proglacial rivers are given in Tables S8 and S9 of the Supporting Information S1. The sample IDs used throughout the discussion correspond to the respective station and sampling depth (refer to Table S1 in Supporting Information S1) and are abbreviated as “Station_Sampling Depth.”

#### 3.2.1 Macronutrients

Macronutrient concentrations exhibited spatial variability across the study area, with surface waters generally having lower concentrations than deeper waters (Figures 3a–3d). Higher nutrient concentrations in intermediate and surface waters of the inner Kongsfjorden indicate that upwelling of nutrient‐rich deep waters occurred. Our results are in good agreement with Halbach et al. (2019), who identified subglacial discharge‐induced upwelling at the Kronebreen‐Kongsbreen system in addition to wind‐induced upwelling as a main pathway to replenish surface waters of inner Kongsfjorden with nitrate and phosphate. In contrast, nitrate and nitrite concentrations at surface station T_10 decreased below LOD and showed minimum concentrations for silicate (0.58 μmol · L^−1^) and phosphate (0.16 μmol · L^−1^). The depletion of surface waters in the outer fjord likely results from primary production combined with the formation of a stable stratification that restricts the exchange of deep waters with higher nutrient concentrations and surface waters. We found maximum nutrient concentrations in the deep waters of station T, F, and PN (Table S8 in Supporting Information S1), which are likely remnants of the previous winter season as we identified these deeper water masses as LW, which is formed during autumn and winter, and TAW as a mixture of LW with AW (Figure 2c). Along the fjord axis, we observed higher nutrient concentrations in the surface waters of inner stations PN and F than those of outer station T, indicating nutrient inputs by glacial discharge from the Kronebreen‐Kongsbreen system. Overall, our results confirm that nutrients in Kongsfjorden are transported by terrestrial freshwater sources (Santos‐Garcia et al., 2022) and largely driven by upwelling at the glacier front (Halbach et al., 2019).

We observed high silicate concentrations in surface waters of station PN and F (Figure 3c) and in river water of proglacial streams, varying between 4.76 μmol · L^−1^ (BR) and 7.44 μmol · L^−1^ (AL), whereas nitrate, nitrite, and phosphate concentrations in river water were significantly lower (refer to Table S8 in Supporting Information S1). This is in line with a study by Santos‐Garcia et al. (2022), who found higher nutrient concentrations at the glacier front with terrestrial sources being enriched in silicate relative to nitrate. Additionally, previous studies showed that glacial meltwater largely exports silicate as labile solid‐phase amorphous silica, which is soluble in downstream marine environments and therefore may represent an important source of dissolved silicate to fjords and near coastal regions (Hatton et al., 2021; Hawkings et al., 2017). Another process controlling dissolved silicate concentrations is the dissolution of sedimentary biogenic silica at the sediment‐water interface (Ma et al., 2023; Ng et al., 2020). We suggest that the dissolution of these labile particulate silicate phases is responsible for increasing silicate concentrations in deep waters. We found a decoupling between the distributions of silicate and the other nutrients, likely because of silicate enrichment relative to nitrate in terrestrial sources and the dissolution of particulate silicate as an additional benthic source.

#### 3.2.2 Carbonate System

In this study, the distribution of AT showed a positive linear correlation with salinity (*R* = 0.99, refer to Figure S2a in Supporting Information S1) with relatively low surface water concentrations, with a minimum of 2,150 μmol · kg^−1^ at the Kronebreen‐Kongsbreen front, where we measured a minimum salinity of 32.07 (Table S8 in Supporting Information S1). Along the salinity gradient, AT concentrations increased, reaching a maximum value of 2,320 μmol · kg^−1^, corresponding to a maximum salinity of 34.91 at the intermediate depths of station T, which we identified as AW. Previous studies of Kongsfjorden showed a similar trend between AT and salinity (Hopwood et al., 2020; Koziorowska‐Makuch et al., 2023), from which we can determine that the AT distribution is mainly controlled by mixing of water masses with different salinities. Another indicator of changes in the marine carbonate system is the calcium carbonate saturation state (Ω), which shows the dissolution potential for solid CaCO_3_. When Ω < 1, waters are undersaturated with respect to CaCO_3_, with CaCO_3_ minerals becoming prone to dissolution (Middelburg, 2019). In surface waters of Kongsfjorden, Ω values were supersaturated with a value of 1.92 at station PN_3, increasing with distance from the Kronebreen‐Kongsbreen system to 2.47 at station T_10 (Table S8 in Supporting Information S1). The spatial variation of AT and Ω in surface waters can be explained by the dilution of seawater by glacial discharge from the Kronebreen‐Kongsbreen system with low carbonate ion concentrations (Hopwood et al., 2020). The surface waters reveal low AT concentrations and therefore exhibit reduced buffering capacities, with pH values appearing rather stable throughout. This can be seen along the fjord axis, where little change in surface water pH from the front of the glacier (station PN_3, pH = 8.21) to the fjord mouth (station T_10, pH = 8.26) occurred (Table S8 in Supporting Information S1). Our findings are in good agreement with previous studies of the carbonate system in Kongsfjorden, where similar pH and Ω ranges (pH 8.13–8.27, Ω 1.5–2.5) were measured in surface waters of the fjord (Koziorowska‐Makuch et al., 2023). In surface waters, biological processes remove CO_2_ from solutions, making them a sink for atmospheric CO_2_. In deep waters, organic matter (OM) decomposition returns CO_2_ to the solution. The distribution of CT thus deviates from a strictly linear correlation with salinity (*R* = 0.91, refer to Figure S2b in Supporting Information S1), rather revealing a nutrient‐type distribution. This is also reflected by low pCO_2_ values in surface waters (222–240 μatm) that increased with depth (254–285 μatm) (Table S8 in Supporting Information S1). This suggests that photosynthetic CO_2_ uptake seasonally mitigates reduced buffering capacities in fjord waters caused by the addition of glacial meltwater from the Kronebreen‐Kongsbreen system and proglacial rivers. By reducing CT and pCO_2_, and increasing pH through primary production, an increase in fjord water acidification is attenuated (Fransson et al., 2016; Hopwood et al., 2020; Middelburg et al., 2020).

Koziorowska‐Makuch et al. (2023) showed that multiple freshwater sources have different AT endmembers but generally dilute AT concentrations in surface waters of Kongsfjorden. However, high erosion rates in the catchment of land‐terminating glaciers continually expose carbonate minerals (Hindshaw et al., 2016), which leads to a larger buffering capacity in freshwater in contact with carbonate and silicate‐rich bedrock (Fransson et al., 2015). Within the Kongsfjorden area, the bedrock typically comprises clastic sediments (sandstone and shale) with intercalated carbonate layers (Dallmann, 2015), with calcite dissolution being the dominating weathering reaction in young proglacial environments due to its higher dissolution rate compared to silicates (Hodson et al., 2002). In our study, we measured AT values varying between 1,261 μmol · kg^−1^ (AL) and 1,305 μmol · kg^−1^ (BR), and CT values ranging from 953 μmol · kg^−1^ (AL) to 1,031 μmol · kg^−1^ (ML) in the freshwater runoff of proglacial rivers (Table S8 in Supporting Information S1). The ratios of AT/CT were high and ranged between 1.23 (ML) and 1.35 (BR). Similar high AT/CT ratios were found by Cantoni et al. (2020) at the mouth of the rivers with 1.21 (ML), 1.24 (BR), and 2.50 (AL). Furthermore, river water was in a similar pH range as fjord water with pH values ranging from 8.01 (BR) to 8.43 (AL), respectively. A long‐term monitoring study of BR showed increasing yearly mean pH values ranging from 7.0 in 1991/1992 to 7.9 in 2010, indicative of increasing chemical weathering (Nowak & Hodson, 2014). A comparative study of freshwater tributaries into Kongsfjorden found a pH value of 6.81 in ML and 8.47 in BR (AL not available) (Cantoni et al., 2020). This suggests that water from proglacial catchments is well buffered and has a low sensitivity toward changes in pH and pCO_2_, which provides a partly negative feedback (or less positive feedback) on further OA (Fransson et al., 2015). Our data suggest that intensive carbonate weathering has taken place in the catchment areas, yielding high AT and CT concentrations in proglacial river water. We hypothesize that besides biological mitigation, the influx of additional carbonates through proglacial streams of land‐terminating glaciers attenuates pH changes due to freshwater dilution by discharge from marine‐terminating glaciers. How different weathering processes of land‐ and marine‐terminating systems influence AT and CT endmembers is further discussed in Section 3.4.

#### 3.2.3 Dissolved Iron (dFe)

Vertical profiles of dFe in Kongsfjorden showed a typical distribution of a recycled trace element with low dFe concentrations in surface waters that increased toward deeper waters (Figure 4a). Previous studies suggest that glaciomarine sediments in Kongsfjorden provide significant benthic fluxes of dFe to the water column that can potentially support the transport of bioavailable Fe to the continental shelf (Herbert et al., 2021; Laufer‐Meiser et al., 2021; Wehrmann et al., 2014). Hereby, dissimilatory metal reduction of Fe(III) oxide‐hydroxide phases and the reaction of these oxide phases with hydrogen sulfide release dFe into porewaters, which is subsequently transported to fjord bottom waters by diffusion and to the upper water column by vertical mixing (Herbert et al., 2021; Wehrmann et al., 2014, 2017). This hypothesis is in good agreement with our findings as deeper waters exhibited higher dFe concentrations than surface waters (Figure 4a). Overall, within the Kongsfjorden drainage basin, we found relatively high dFe concentrations in proglacial rivers ranging between 348 ± 3 ng · L^−1^ and 7,400 ± 300 ng · L^−1^ for BR and AL, respectively (Table S9 in Supporting Information S1). The large range of dFe concentrations in the proglacial streams is probably due to flocculation at the sampling location of BR. Zhang et al. (2015) found that around 80% of dFe in BR is lost during the early stages of estuarine mixing. When comparing the dFe concentration in the proglacial rivers with the average value found in Kongsfjorden surface waters of 188 ng · L^−1^, we found an estuarine removal factor of 46%–97%. This high removal factor emphasizes the strong impact of flocculation on the transport of dFe from terrestrial sources to coastal waters (Bruland et al., 2014; Klunder et al., 2012) and is in good agreement with losses of up to 90% calculated by Zhang et al. (2015). Although significant amounts of dFe are removed during estuarine mixing, we agree with Shen et al. (2024) who found glacial meltwater to be a primary source for dFe in Kongsfjorden. During flocculation, reactive particulate Fe is produced that can be reduced in the fjord sediments, providing an additional benthic source of dFe (Herbert et al., 2022; Zhang et al., 2015). Overall, we found the distribution of dFe to be influenced by subglacial and riverine freshwater input, the strong removal during estuarine mixing, biological processes that induce recycling in the water column, and benthic input due to early diagenetic processes in the sediments.

#### 3.2.4 Dissolved Manganese (dMn)

In our study, we report a surface dMn maximum near Kronebreen glacier of 3,180 ± 60 ng · L^−1^ (PN_3; SW) and an intermediate depth minimum in the outer part of the fjord of 330 ± 6 · ng L^−1^ (T_137; AW) (Table S9 in Supporting Information S1 and Figure 4b). Yang et al. (2022) found the distribution of dMn in Kongsfjorden to be mainly controlled by mixing of SW with high dMn concentrations from glacial meltwater, and AW with low dMn concentrations. Furthermore, our results show increasing dMn concentrations toward the bottom waters of all stations (Figure 4b). In line with previous studies, we suggest that resuspension and a flux of benthic dMn from sediments are an explanation for elevated deep water concentrations (Wehrmann et al., 2014; Yang et al., 2022). Similar to dFe, porewaters of Kongsfjorden sediments were found to exhibit elevated dMn concentrations, resulting from the reduction of manganese oxide phases (Herbert et al., 2021; Wehrmann et al., 2014), which can be exported to fjord bottom waters by diffusion and returned to the upper water column by vertical mixing (Wehrmann et al., 2014). Another important source of dMn to Kongsfjorden are proglacial rivers where we found concentrations ranging from 6,850 ± 250 ng · L^−1^ to 22,000 ± 600 ng · L^−1^ for AL and ML, respectively (Table S9 in Supporting Information S1). In the outlet of BR, we measured dMn concentrations of 9,710 ± 210 ng · L^−1^, which is lower than what Yang et al. (2022) reported at a similar location (13,400 ± 500 ng · L^−1^). Temporal differences in dMn concentrations of BR between the two studies are likely explained by a varying discharge volume from Austre Brøggerbreen, inflow from tributaries, and groundwater sources. Considering the importance of particulate Mn and Fe in removing other trace elements from surface waters (Goldberg, 1954), we hypothesize that the amount of dMn and dFe released by terrestrial runoff and their transformation into the respective particulate phases during estuarine mixing is closely linked to scavenging of other elements (van Hulten et al., 2017; Xiang et al., 2021) from surface waters of Kongsfjorden. Physical mixing and disturbance of bottom sediments by bioturbation or bottom currents resuspends large amounts of sediment and facilitates desorption from particles (de Souza Machado et al., 2016), making bottom sediments a secondary source for trace elements (Herbert et al., 2020).

#### 3.2.5 Dissolved Cadmium (dCd)

The vertical profiles of dCd in Kongsfjorden showed higher surface water concentrations at inner stations F, IA, and PN than at outer station T, with increasing concentrations toward deeper waters (Figure 4c). This is consistent with studies across the Arctic, which found dCd concentrations to be depleted in surface waters and to increase with depth due to the uptake by microorganisms and deep water organic matter remineralization leading to dCd release (Achterberg et al., 2021; Gerringa et al., 2021; Zhang et al., 2019). In this study, linear regression of dCd concentrations against phosphate gave a coefficient of determination of *R* = 0.90, highlighting the strong positive relationship between dCd and phosphate in Kongsfjorden (refer to Figure S2c in Supporting Information S1). Previous studies of the Arctic and Eastern Atlantic surface and deep waters (Pohl et al., 1993), and the Chukchi Sea continental shelf and slope region (Kondo et al., 2016) demonstrated that the distribution of dCd is generally controlled by internal biogeochemical cycles that also affect phosphate (Cid et al., 2012; Kondo et al., 2016). Compared to fjord water, proglacial rivers showed low dCd concentrations ranging between 1.9 ± 0.28 ng · L^−1^ (ML) and 5.04 ± 0.23 ng · L^−1^ (AL) (refer to Table S9 in Supporting Information S1), suggesting a minor contribution of proglacial rivers to dCd concentrations in Kongsfjorden. Gerringa et al. (2021) also found little riverine supply to seawater of the Nansen Basin, suggesting that dCd distribution does not vary much due to river input. Because of the strong relationship with phosphate and low dCd concentrations found in proglacial rivers, we suspect upwelling of deeper waters with higher dCd concentrations to supply surface waters of the inner fjord stations. Since the primary source of dCd to porewaters is early diagenesis of OM (de Baar et al., 1994; Smrzka et al., 2019), elevated concentrations found in overlying waters might indicate efficient OM remineralization in surface sediments of Kongsfjorden (Herbert et al., 2022).

#### 3.2.6 Dissolved Vanadium (dV)

We observed low surface water dV concentrations at the inner stations (Figure 4d) with minimum concentrations found at the glacier‐adjacent station PN with a value of 1,170 ± 40 ng · L^−1^. Compared to the fjord waters, riverine concentrations of dV were very low, ranging between 29.8 ± 0.5 ng · L^−1^ (BR) to 54.0 ± 1.6 ng · L^−1^ (ML) (Table S9 in Supporting Information S1). Low dV concentrations in freshwater were also found by Whitmore et al. (2019) in sea ice melt and riverine inputs across the Western Arctic Ocean. We attribute the distribution of dV in Kongsfjorden to be mainly affected by freshwater supply from marine‐ and land‐terminating glaciers, thus diluting the seawater dV signal in surface waters of Kongsfjorden. Another reason for decreasing dV concentrations in surface waters is likely particle interactions in the meltwater plume released by the Kronebreen‐Kongsbreen system that remove dV from the water column. This is supported by the study by Whitmore et al. (2019), who found that dV was significantly removed in estuarine and shelf environments of the Western Arctic Ocean. Here, they argued that scavenging by Fe(III) and Mn(III/IV) oxide‐hydroxide and delivery to the sediments is likely to be the dominant factor for dV removal (Smrzka et al., 2019; Whitmore et al., 2019).

#### 3.2.7 Dissolved Lead (dPb)

Vertical dPb concentration profiles show that surface concentrations (17.8 ± 0.4 ng · L^−1^; F_6) were significantly elevated in comparison to intermediate water concentrations (2.43 ± 0.28 ng · L^−1^; T_73) at stations PN, F, and IA (Table S9 in Supporting Information S1). This signifies that terrestrial freshwater runoff from land‐ and marine‐terminating glaciers is a major source of dPb to the fjord. In the outer fjord, low dPb concentrations in the water column of station T (Figure 4e) indicate that dPb is removed from the dissolved phase by scavenging and association of dPb with particulate Mn and Fe (Achterberg et al., 2021; Bruland et al., 2014). In the inner fjord area, dPb concentrations in surface waters showed an unusual distribution with similar concentrations at the inner fjord station PN and the mid fjord station F. We attribute this to the progressive cycling of Pb between the dissolved and (labile) particulate phases. As sediment load declines along the fjord axis, glacier‐derived particles can transition from a net sink to a net source of dPb, resulting in high surface water concentrations (Krause et al., 2023). In addition to the surface waters of stations PN, IA and F, the deep waters of PN also showed increased dPb concentrations, suggesting dPb release by remobilization from sediments promoted by upwelling. This is in agreement with higher Pb values in marine sediments at the inner fjord (Choudhary et al., 2020), which could act as a secondary source when disturbed by upwelling. The dPb concentration in proglacial rivers varied greatly, ranging between 4.1 ± 0.3 ng · L^−1^ (ML) and 98 ± 7 · ng L^−1^ (AL), with concentrations below LOQ for BR (<1.9 ng · L^−1^; refer to Table S9 in Supporting Information S1). We suggest differences in bedrock composition of the Kongsfjorden catchment area to influence spatial variations of dPb concentration in proglacial rivers, as shown by Kozak et al. (2016), in a study of the Revelva catchment located in the Wedel‐Jarlsberg Land, in the southern part of Svalbard. They attributed the amount of precipitation, the geochemical background of the bedrock, and dust deposition from long‐range transport of both natural and anthropogenic origin to be responsible for temporal variability in dPb concentrations (Kozak et al., 2016). In general, elevated dPb concentrations in the high‐latitude North Atlantic are attributed to anthropogenic Pb inputs (Achterberg et al., 2021) resulting from the former use of leaded gasoline (Bruland et al., 2014) and other industrial emissions through long‐range transport (Pohl et al., 1993; Stalwick et al., 2023). A recent study measured Pb isotopic ratios in sediment cores from Kongsfjorden and found that the isotopic composition indicates a very high proportion of natural Pb in the particulate phase (Zaborska et al., 2017). Without further isotopic analysis, we cannot distinguish between natural and anthropogenic sources of dPb in the fjord water of Kongsfjorden. However, given the natural origin of particulate Pb in sediment records (Zaborska et al., 2017), we suggest that the main source of dPb is weathering of glacier bedrock, which then becomes part of the particulate phase through scavenging.

### 3.3 Influences and Contributions of Physicochemical Processes on the Distribution of Carbon, Nutrients, and Trace Elements in Kongsfjorden

In this study, we used multivariate statistical methods to process a complex Kongsfjorden data set composed of multiple interconnected parameters. This allowed us to gain a deeper insight into the physicochemical processes that alter the distribution of carbon, nutrients, and trace elements in the coastal waters of Kongsfjorden discussed in Section 3.2. We used PCA to identify the dependencies among the parameters that we can attribute to physicochemical processes. Based on PC loadings, we used a cluster analysis to disclose similar trends or identical sources among the investigated parameters. Each parameter was assigned to a specific cluster based on its maximum similarity to the other parameters.

**Figure 5:**
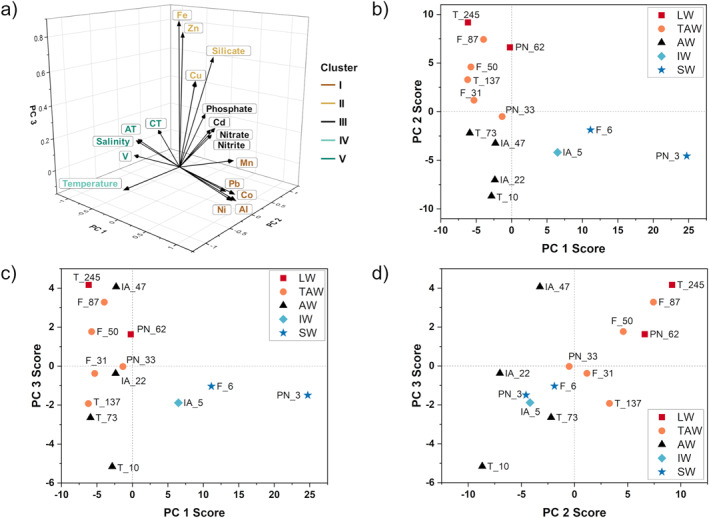
(a) The spatial distribution of PC loadings, including the results of the subsequent cluster analysis, is indicated by color codes. Score plots according to PCA results are shown for (b) PC 1 score versus PC 2 score; (c) PC 1 score versus PC 3 score; and (d) PC 2 score versus PC 3 score, with colors indicating the water mass of the sample.

The results of the PCA are summarized in Table 1. Overall, 88% of the total variance in the standardized data set can be explained by 3 PCs. The majority of variance is explained by PC 1 with 45%, followed by PC 2 with 29%, and PC 3 with 14%. The corresponding Eigenvalues are 8.6, 5.2, and 2.4, respectively. In Table 1, significant PC loadings of each parameter are printed in bold. Loadings close to −1 or 1 indicate a strong correlation between the parameter and the PC, with the algebraic sign of a loading being arbitrary. With the exception of silicate, which loads significantly on both PC 2 and PC 3, all parameters significantly load on one PC. Multiple parameters with high loadings on the same PC usually indicate that they were influenced by the same physicochemical process. Figures 5b–5d show the PC score plots with colors indicating the water mass of the sample. In general, the score plots show a clear spatial separation of our samples according to the respective water mass. The cluster analysis revealed a total of five clusters that are shown in Figure 5a as a 3D diagram of the spatial distribution of each parameter's PC loading with colors indicating the cluster affiliation.

**Table 1:** Loadings of Varimax Rotated Principal Components (PCs) of 18 Parameters From the Dissolved Fraction of Kongsfjorden Water Column

Parameter	PC 1 conservative mixing and freshwater input			PC 2 pelagic processes and water masses			PC 3 benthic processes		
	Loading	$R_{S}$ (%)	*P*	Loading	$R_{S}$ (%)	*P*	Loading	$R_{S}$ (%)	*P*
Salinity	**−0.98**	80	***	0.10	20	***	0.07	–	–
Temperature	−0.25	44	***	**−0.90**	56	***	−0.06	–	–
NO_3_^−^	−0.15	38	***	**0.96**	62	***	0.09	–	–
NO_2_^−^	−0.18	38	***	**0.97**	62	***	0.12	–	–
Si(OH)_4_	0.13	41	***	**0.59**	–	–	**0.65**	59	***
PO_4_^3−^	−0.25	35	***	**0.91**	65	***	0.23	–	–
AT	**−0.97**	84	***	0.13	16	**	0.07	–	–
CT	**−0.85**	37	***	0.48	55	***	0.11	8	·
dAl	**0.99**	75	***	−0.07	25	*	−0.09	–	–
dV	**−0.98**	100	***	0.05	–	–	−0.03	–	–
dFe	−0.21	–	–	0.23	41	**	**0.87**	59	***
dMn	**0.95**	70	***	−0.02	–	–	0.14	30	**
dCo	**0.99**	72	***	−0.02	28	***	−0.06	–	–
dNi	**0.98**	70	***	0.03	30	*	−0.11	–	–
dCu	0.55	–	–	−0.34	55	**	**0.60**	45	**
dZn	−0.17	–	–	0.26	39	·	**0.80**	61	**
dCd	−0.06	39	***	**0.93**	61	***	0.15	–	–
dPb	**0.80**	100	**	0.05	–	–	−0.07	–	–

*Note*. Significant loadings, according to the broken stick analysis, are shown in bold. The importance of physicochemical processes identified by principal component analysis is given as the proportion (%) of process‐related change to overall change in distribution (R_S_) and the significance of the contribution (*P*). The results of the multiple linear model, with the significance of contributions of physicochemical processes identified by principal component analysis on overall change in distribution for each parameter, are indicated as follows: (“***”: *P* < 0.001; “**”: *P* < 0.01; “*”: *P* < 0.05; and “·”: *P* < 0.1, with a cutoff value of *P* ≥ 0.1). Contributions not accounted for by PCA are not regarded, as they are negligibly small.

Quantification of physicochemical processes identified with PCA was carried out using an adapted APCS‐MLR analysis. According to Appendix A, Equation A11, we can use multiple linear regression to model the distribution of each parameter depending on the physicochemical processes identified by PCA. This allows for a better estimation of the importance of each physicochemical process (proportional contribution) on each parameter's distribution in Kongsfjorden. A summary of the APCS‐MLR results is given in Table 1, which shows the contribution of each individual physicochemical process $R_{S}$ compared to the other processes. Additionally, we report the significance *P* of the contribution made by each physicochemical process on the distribution of each parameter in the water column. Considering the limited size of the data set, we might not be able to statistically detect the influence of certain processes on the distribution of specific elements. This does not mean that the influence is non‐existent, but rather that the influence of other processes is so strong that minor changes become insignificant and are disregarded by the statistical model.

#### 3.3.1 Conservative Mixing and Freshwater Input

The high loading of salinity on PC 1 suggests that PC 1 resolves the salinity gradient of Kongsfjorden. This indicates for a conservative mixing behavior of the other parameters with high loadings on PC 1 (AT, CT, dAl, dV, dMn, dCo, dNi, and dPb) (Table 1). As seen in the score plots in Figures 5b and 5c, PC 1 decomposes the samples into surface water observations (high scores) versus intermediate to deep water observations (low scores). We suggest that PC 1 is an indicator of freshwater supply, as SW is influenced by terrestrial freshwater runoff and separated from other water masses by a strong halocline (refer to Section 3.1). The result of the cluster analysis shows that for parameters loading significantly on PC 1, two groups can be identified: salinity, AT, CT and dV as Cluster V and dAl, dMn, dCo, dNi, and dPb as Cluster I. Spatially, these two clusters are located on opposite sides along the axis of PC 1 (Figure 5a). The highest PC 1 score is obtained for shallow water samples (PN_3, followed by F_6 and IA_5), with all other samples having a negative PC 1 score. This spatial differentiation, combined with the assignment into two opposing clusters, indicates that PC 1 is resolving terrestrial freshwater supply either as a source for measured parameters (dAl, dMn, dCo, dNi, dPb) or is responsible for decreasing concentrations (salinity, AT, CT, dV) through dilution.

The decreasing PC 1 scores of surface water observations along the fjord axis from PN_3 and F_6 to T_10 suggest that the relative distance of the station from the freshwater source plays an important role in fjord distributions (Figures 5b and 5c). As the lateral distance from the Kronebreen‐Kongsbreen system increases, the influence of the glacial runoff on the distribution of parameters with high loadings on PC 1 decreases. A similar conclusion can be reached by looking at the effect of the specific freshwater content of each station on fjord distributions. As previously described, surface waters of station IA receive less direct discharge from the Kronebreen‐Kongsbreen system because of the predominant current system, which is also expressed in a lower PC 1 score for IA_5 compared to the other surface water samples from the inner fjord (Figures 5b and 5c). We thus suggest that not only the lateral distance from the freshwater source but also the current system affects fjord distributions of parameters identified by PC 1. This finding corresponds well with results from a study that compared six contrasting glacier fjords around Greenland and showed that salinity and lateral distance from areas of strong glacier influence impacted trace metal distribution (Krause et al., 2021).

Quantitatively, terrestrial freshwater influx and conservative mixing (PC 1) are the most important physicochemical processes for the majority of the parameters (salinity, AT, dAl, dV, dMn, dCo, dNi, and dPb), contributing between 70% (dMn, dNi) to 100% (dV, dPb) to the overall change in the parameter's distribution compared to the other processes (Table 1). This finding suggests that freshwater influx and mixing with fjord water is the dominant factor in changing the distribution of almost all parameters. This is consistent with the depth profiles of these parameters as seen in Figures 3 and 4 and Figure S1 in Supporting Information S1.

#### 3.3.2 Pelagic Processes and Water Mass Distribution

The allocation of parameters that load significantly on PC 2 (temperature, nitrate, nitrite, silicate, phosphate, dCd) suggests that PC 2 describes pelagic processes, such as biological cycling controlled by organic matter production, sinking and remineralization in the water column, and water mass distributions (Table 1). Quantitatively, this is indicated by higher $R_{S}$ values ranging from 56% (temperature) to 65% (phosphate) for PC 2. Parameters with high PC 2 loading are distributed on opposite sides of the PC 2 axis (Figure 5a). This can be seen in the cluster analysis, which distinguishes two clusters: Cluster III, a nutrient grouping, with nitrate, nitrite, phosphate, and dCd, and Cluster IV with temperature. The opposing trends of the temperature and nutrient profiles can be attributed to the intrusion of warmer, and nutrient‐depleted AW into subsurface waters alongside colder, and nutrient‐rich LW in deep waters (refer to Section 3.1, Figures 2c and 3a–3d). The strong biological influence on the distribution of dCd was discussed in Section 3.2.5.

As shown in the score plot of PC 2 (Figures 5b and 5d), PC 2 further differentiates between surface and subsurface samples (AW, IW, and SW) with low PC 2 scores, and intermediate and deep water samples (LW and TAW) with higher PC 2 scores. The higher PC 2 scores at stations PN and F indicate that upwelling at the Kronebreen‐Kongsbreen system affects the distribution of parameters, leading to more nutrient‐rich waters in the inner parts of the fjord (Halbach et al., 2019). In summary, fjord distributions of parameters with high loadings on PC 2 are driven by pelagic processes, that is, internally by biological cycling, and externally by the presence or absence of specific water masses (AW intrusion) and upwelling at the glacier terminus.

We can also identify the importance of PC 2 for changes in the distributions of dAl, dFe, dCo, dNi, dCu, dZn, and dCd as $R_{S}$ values range between 25% (dAl) and 61% (dCd). This is in line with the depth profiles (Figure 4 and Figure S1 in Supporting Information S1), which show a gradual increase in concentration with depth. We assume that these elements are removed from surface waters via assimilation and incorporation into, or adsorption onto, OM and returned to the water column as OM sinks and is remineralized. A biological influence on trace elements in the Arctic environment was found in multiple studies for dAl (Middag et al., 2009), dFe (Klunder et al., 2012), dCo (Bundy et al., 2020; Hawco et al., 2018), dNi (Jensen et al., 2022) dCu (Gerringa et al., 2021), dZn (Jensen et al., 2019; Middag et al., 2019), and dCd (Zhang et al., 2019). An interesting finding is the difference in $R_{S}$ values of PC 2 for AT (16%) and CT (55%), which can be explained by their different behavior during biological processes (Table 1). During photosynthesis, dissolved CO_2_ is incorporated by organisms and converted to particulate and dissolved organic carbon, which decreases CT concentrations in surface waters. During respiration and decomposition of organic material, CO_2_ is released back into the water, increasing CT concentrations in deeper waters. Changes in AT distribution indicated by PC 2 occur due to nitrate‐based primary production, which is a source for AT, while aerobic respiration accompanied by nitrification represents an alkalinity sink (Goldman & Brewer, 1980; Middelburg et al., 2020). Since AT is influenced indirectly by biological processes, the impact that PC 2 has on the AT distribution is less pronounced than for CT, which is directly cycled as a nutrient.

#### 3.3.3 Benthic Processes

For PC 3, we suggest that benthic processes in surface sediments (i.e., reversible scavenging linked to mineral dissolution and remineralization of organic material) influence the distribution of parameters with high PC 3 loadings (silicate, dFe, dCu, and dZn). While PC 2 separates the observations according to water masses, PC 3 decomposes according to water depth, with PC 3 scores following the depth profile of each station (Figures 5c and 5d). We attribute this increase in PC 3 scores to deep water with elevated concentrations due to element release from bottom sediments, which is also reflected in the depth profile of parameters with high loadings on PC 3 (Figures 3c and 4a and Figures S1d and S1e in Supporting Information S1). We note the linearity between PC 2 and PC 3 (Figure 5d), which we relate to the similar impact of both processes on parameter distributions with an underlying dependency on depth. Both processes lead to increasing concentration with depth; however, while PC 2 explains the internal mixing and recycling of constituents, PC 3 illustrates the contribution of external sources, such as mineral particles and surface sediments.

The cluster analysis assigns all parameters that load significantly on PC 3 to Cluster II (Figure 5a). Interestingly, silicate is not assigned to Cluster III together with the other nutrients, even though it loads significantly on PC 2. Additionally, silicate has a (small) positive loading on PC 1, whereas the other nutrients have a negative PC 1 loading (Table 1). Quantitatively, changes in the distribution of silicate are mostly driven by PC 1 (41%) and PC 3 (59%). This further highlights the decoupling between the distributions of silicate and the other nutrients because of silicate enrichment relative to nitrate in freshwater (Santos‐Garcia et al., 2022) and the dissolution of labile particulate silicate phases at the sediment‐water interface (Hatton et al., 2021; Hawkings et al., 2017; Ma et al., 2023).

Looking more closely at the distribution of PC 3 scores (Figure 5d), we noticed an exceptionally high score for sample IA_47, which is also reflected by high concentrations in the depth profiles of silicate, dFe, dCu, and dZn (Figures 3c and 4a, Figures S1d and S1e in Supporting Information S1). This finding agrees with Herbert et al. (2021), who found the highest benthic Fe fluxes located in mid‐Kongsfjorden, which they attribute to an ideal balance between the delivery of reactive glacial flour and sufficient labile organic carbon to drive enhanced Fe fluxes. Overall, biotic and abiotic redox reactions in the sediment of Kongsfjorden have been shown to supply the fjord bottom water with dFe (Herbert et al., 2021; Laufer‐Meiser et al., 2021) and dMn (Wehrmann et al., 2014), for which we found high $R_{S}$ values for PC 3 of 59% (dFe) and 30% (dMn) (Table 1). A contribution of PC 3 to changes in the distribution of dCu (45%) and dZn (61%) was also found by our study (Table 1). Similarities between the distribution of dZn and silicate have been shown in previous studies (Achterberg et al., 2021; Middag et al., 2019), suggesting a sediment remineralization source of dZn to deep waters concurrent with labile amorphous silica (Jensen et al., 2019). Benthic sources have also been proposed as significant inputs of dCu to bottom waters, with dCu regenerated through decomposition of biogenic particles or reversible scavenging at the sediment‐water interface (Jensen et al., 2022; Takano et al., 2014). In Kongsfjorden, we suggest that an interplay of dissolution and redox reactions in the sediment drives benthic fluxes of silicate, dFe, dMn, dCu, and dZn, likely providing additional sources of these elements to the water column of Kongsfjorden.

Besides their different behavior during biological cycling (Section 3.3.2), AT and CT are also impacted differently by benthic processes. This can be seen in the $R_{S}$ value of PC 3, which gives a value of 8% for CT and is of no significance for AT (Table 1). Alkalinity generation in porewaters is largely dominated by net sulfate reduction, which contributes little to the effective alkalinity flux to the water column, because the burial of reduced sulfur is small and is almost entirely reoxidized in the water column (Krumins et al., 2013). This is in good agreement with our results and those of Koziorowska‐Makuch et al. (2023), who found AT fluxes from sediments to the bottom water of Kongsfjorden to be rather insignificant. In contrast, CT fluxes from sediments are largely sustained by benthic organic carbon degradation, which produces 1 mol of CT for each mole of organic C that is oxidized (Krumins et al., 2013), explaining the impact of processes at the sediment‐water interface (PC 3) on the distribution of CT that we see in our results.

### 3.4 Quantification of Freshwater Endmembers

Following the previous assumption that PC 1 describes the influence of conservative mixing along the salinity gradient, we can derive a freshwater endmember (salinity = 0) for subglacial discharge from the Kronebreen‐Kongsbreen system and a high salinity endmember (salinity = 34.86), which is assumed to be composed of the water masses AW, TAW, and LW. As shown in Appendix A, Equation A5, the original data matrix [Z] can be considered as a linear combination of PC scores [P] with PC loadings [B] as their coefficients. For parameters significantly loading on PC 1, we can calculate partial values that are solely influenced by PC 1 by multiplying the loading of the associated variable ${\left[B\right]}_{\text{PC}1}$ with the PC score of the observation ${\left[P\right]}_{\text{PC}1}$. Each partial value was de‐standardized and plotted against salinity to give robust endmember relationships, which we defined through linear regression (refer to Figure S3 in Supporting Information S1). This method was described previously by Mears et al. (2020) to calculate endmembers in the Canadian Arctic Archipelago. The river endmember is given as the mean value of the terrestrial stations BR, ML, and AL (refer to Tables S8 and S9 in Supporting Information S1) with combined uncertainties.

The results of using PC 1 as a singular impact on the data to derive endmembers for this area are shown in Table 2. This procedure suppresses the effect of PC 2 and PC 3 on the data and enables us to find freshwater (*S* = 0) and high salinity endmembers (*S* = 34.86) through linear regression of the modified data against salinity. Considering the limited number of samples available for this study, we can only provide a general overview of endmember estimates. Especially for the trace elements, we want to provide an initial informative basis for future research.

**Table 2:** Concentrations of AT, CT, dAl, dV, dMn, dCo, dNi, and dPb for Kronebreen‐Kongsbreen, Proglacial River and High Salinity Endmembers

Parameter	Kronebreen‐Kongsbreen endmember (*S* = 0)	Mean proglacial river endmember	High salinity endmember (*S* = 34.86)	Literate values for open ocean concentrations
AT (μmol · kg^−1^)	182	1,280 ± 20	2,319	2,308^a^
CT (μmol · kg^−1^)	217	980 ± 30	2,120	2,074^a^
dAl (ng · L^−1^)	46,300	26,000 ± 15,000	86	8–1,100 (55)^b^
dV (ng · L^−1^)	−6,900 *	38 ± 12	1,800	1,570–1,930 (1,830)^b^
dMn (ng · L^−1^)	33,600	13,000 ± 7,000	595	750 ± 170^c^
dCo (ng · L^−1^)	1,400	30 ± 15	12	0.18–18 (2.4)^b^
dNi (ng · L^−1^)	2,500	126 ± 70	230	120–720 (480)^b^
dPb (ng · L^−1^)	226	51 ± 47	5	0.85–32 (2.1)^b^

*Note*. Kronebreen‐Kongsbreen and high salinity endmembers were calculated by linear regression of PC 1‐derived partial values against salinity. *Negative dV endmember as an artifact of the statistical analysis; explained in more detail in the text. Proglacial river endmembers are given as the mean value of terrestrial stations BR, ML, and AL with standard deviations (U (*k* = 1)). Literature values for trace elements are given as the range of concentrations observed in the open ocean, with an estimate of the element's mean concentration in brackets.

^a^ References of literature values: Koziorowska‐Makuch et al. (2023).

^b^ Bruland et al. (2014).

^c^ Yang et al. (2022).

The values that we estimate to be the AT and CT Kronebreen‐Kongsbreen freshwater endmembers are 182 μmol · kg^−1^ (AT) and 217 μmol · kg^−1^ (CT), respectively. These values correspond well with AT (174 μmol · kg^−1^) and CT (247 μmol · kg^−1^) estimated endmembers for the Cumberland Sound on the east coast of Baffin Island (Turk et al., 2016). As it is nearly impossible to directly sample and measure subglacial discharge, estimated AT and CT endmembers are useful indicators for hydrogeochemical models to further understand potential changes in the carbonate system. When comparing the mean endmember of the proglacial rivers with the endmember of the Kronebreen‐Kongsbreen system, it becomes apparent that both endmembers are significantly different from one another. The mean AT (1,280 ± 20 μmol · kg^−1^) and mean CT (980 ± 30 μmol · kg^−1^) values of the proglacial rivers are significantly higher than the Kronebreen‐Kongsbreen endmember. We believe that the large differences between AT and CT values of the subglacial and proglacial endmembers are a reflection of the proton source used during weathering. The two main sources of protons in meltwaters were found to be either the dissolution and dissociation of atmospheric CO_2_ (carbonic acid) or microbially mediated sulfide oxidation (sulfuric acid) (Lehmann et al., 2023; Raiswell, 1984; Tranter et al., 1993). The very low AT and CT values of the subglacial endmember indicate that microbially mediated sulfide oxidation dominates weathering (Brown, 2002; Tranter & Wadham, 2014). This is in line with a study of the Werenskioldbreen (south‐west Svalbard), which found sulfide oxidation to dominate in the subglacial channels (Stachnik et al., 2016). The high AT and CT values of the mean proglacial river endmember correspond well with a multiannual study of the meltwater hydrochemistry of the Bayelva River that found carbonate weathering with carbonic acid to be the dominant weathering process in this proglacial system (Nowak & Hodson, 2014).

For the high salinity endmember, we estimate 2,319 μmol · kg^−1^ (AT) and 2,120 μmol · kg^−1^ (CT), respectively. These findings correspond well with deep water maxima of 2,308 μmol · kg^−1^ (AT) and 2,074 μmol · kg^−1^ (CT) in Kongsfjorden (Koziorowska‐Makuch et al., 2023). Koziorowska‐Makuch et al. (2023) report an average freshwater AT endmember of 595 ± 84 μmol · kg^−1^ (AT) for the West Svalbard area, emphasizing the complexity of the marine carbonate system in high Arctic fjords due to multiple freshwater endmembers and an intricate hydrological setting.

Yang et al. (2022) studied the distribution of dMn in Kongsfjorden and found the river endmember for Bayelva River (BR) to be 13,500 ± 500 ng · L^−1^, which is in excellent agreement with our mean proglacial river endmember of 13,000 ± 7,000 ng · L^−1^. Overall, they found the influence of riverine influx from BR on the distribution of dMn in the water column of Kongsfjorden to be less significant, since they estimated a much larger Kronebreen‐Kongsbreen endmember of 32,000 ng · L^−1^ (Yang et al., 2022). With our study, we can validate this finding with a Kronebreen‐Kongsbreen dMn endmember of 33,600 ng · L^−1^. Yang et al. (2022) also gave values for dMn in AW of 750 ± 170 ng · L^−1^, which corresponds well with our findings of 595 ng · L^−1^. Our Kronebreen‐Kongsbreen endmember for dAl of 46,300 ng · L^−1^ is also in line with Shen et al. (2024), who gave a glacial dAl endmember of 29,000 ± 5,400 ng · L^−1^ (Shen et al., 2024). To the best of our knowledge, no endmember data for other dissolved elements in this area have been published previously. We report glacial endmembers from Kronebreen‐Kongsbreen of −6,900 ng · L^−1^ (dV), 1,400 ng · L^−1^ (dCo), 2,500 ng · L^−1^ (dNi), and 226 ng · L^−1^ (dPb), respectively. We hypothesize that the negative glacial endmember for dV results from strong scavenging by Fe(III) and Mn(III/IV) oxide‐hydroxide (Whitmore et al., 2019) in the glacial plume, which precludes the statistical model from reporting a plausible dV endmember. For the river endmembers, we found values ranging between 26,000 ± 15,000 ng · L^−1^ (dAl), 38 ± 12 ng · L^−1^ (dV), 30 ± 15 ng · L^−1^ (dCo), 126 ± 70 ng · L^−1^ (dNi), and 51 ± 47 ng · L^−1^ (dPb), respectively. The large uncertainty values of actual measured dissolved elements in rivers indicate that complex factors influence element concentrations in rivers of land‐terminating glaciers, for example, transit time, extent of drainage network, access to atmospheric gases, type of bedrock, and weathering rates (Rutter et al., 2011; Shiller & Boyle, 1987; Wadham et al., 1998). A broader comparison with fjords from different regions of Svalbard would help to identify regional variabilities in the composition of coastal waters, potentially caused by bedrock geology and different glacier types. The high salinity endmembers of these elements are 86 ng · L^−1^ (dAl), 1,800 ng · L^−1^ (dV), 12 ng · L^−1^ (dCo), 232 ng · L^−1^ (dNi), and 5 ng · L^−1^ (dPb), respectively. Literature values for the range of concentrations observed in the open ocean are given in Table 2 and correspond well to our estimated high salinity endmembers.

## 4 Summary and Conclusion

### 4.1 Influence of Marine‐ Versus Land‐Terminating Glaciers

**Figure 6:**
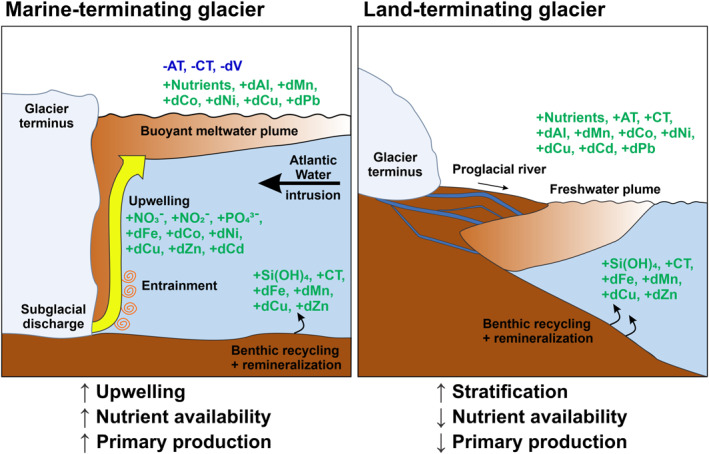
Illustration explaining how different glacier types (land‐ vs. marine terminating glaciers) influence the biogeochemical cycling of carbon, nutrients, and trace elements in Kongsfjorden.

We found that the biogeochemical cycles of Kongsfjorden were influenced by the different chemical compositions of proglacial and subglacial discharges, as well as by physically driven effects triggered by the glacier systems. In Figure 6, we summarized our findings by illustrating the major influences of the two contrasting glacier types on Kongsfjorden. We attribute differences in the chemical signature of the marine‐terminating Kronebreen‐Kongsbreen glacier system from the three land‐terminating glaciers to different weathering regimes in these contrasting glacial environments. Previous studies have shown the importance of mechanochemical processes beneath glaciers for creating fresh reactive mineral surfaces that lead to significant enrichment of dissolved constituents in subglacial discharge (Hatton et al., 2021; Kanna et al., 2018). In front of marine‐terminating glaciers, the upwelling water contains subglacial weathering products (Jørgensen et al., 2021), which we identified as the dominant source for dAl, dMn, dCo, dNi, dCu, and dPb in the surface waters of Kongsfjorden (Figure 6, left panel). In contrast, it has a diluting effect on surface water concentrations of AT and CT, and promotes scavenging of dV in the glacial plume. With decreasing AT concentrations, fjord waters exhibit lower buffering capacities and become more prone to pH changes (Koziorowska‐Makuch et al., 2023), amplifying the effect of OA (Fransson et al., 2015; Hopwood et al., 2020).

When glacial meltwater emerges into proglacial environments, the meltwater equilibrates with the atmosphere and is in prolonged contact with reactive material, making proglacial zones highly geochemically reactive (Wadham et al., 2001). From the freshwater endmember calculation, we found concentrations of dissolved elements in proglacial rivers to be approximately 1–2 orders of magnitude lower than discharge from marine‐terminating glaciers. In terms of carbonate chemistry, we measured high AT and CT concentrations in riverine runoff, which could attenuate the effect of freshwater dilution by subglacial runoff on OA in the surrounding coastal environment. Together, these differences in weathering regimes result in different chemical signatures in proglacial and subglacial discharges, which actively impact biogeochemical cycles in Kongsfjorden.

Our study of three outflows of proglacial catchments from major land‐terminating glaciers shows that the overall freshwater composition between the catchments varies significantly. Spatial variations in individual proglacial rivers are likely explained in terms of bedrock composition, discharge volume, and the rate of chemical and physical weathering (Hindshaw et al., 2016; Rutter et al., 2011). This highlights the importance of selecting multiple sampling sites when studying freshwater characteristics, as the variance in proglacial catchments can be extremely high.

Besides chemical differences in proglacial and subglacial discharge, we found physically driven effects triggered by the different glacier systems to alter biogeochemical cycles. The subglacial discharge at the base of marine‐terminating glaciers creates a highly turbulent zone in the inner part of fjords, which transports nutrients from deep water to the photic zone (Halbach et al., 2019; Hopwood et al., 2018; Meire et al., 2017; Meslard et al., 2018). Previous studies have shown that upwelling induced by subglacial discharge at the Kronebreen‐Kongsbreen glacier front supplies inner Kongsfjorden with nitrate and phosphate (Halbach et al., 2019). We agree with this and suggest that the same process also influences the distribution of nitrite, dFe, dCo, dNi, dCu, dZn, and dCd, which are likely released by OM degradation and subsequently entrained into the photic zone by upwelling (Figure 6, left panel). In contrast, we found lower nutrient availability in areas of land‐terminating glaciers due to less turbulent mixing and a more stratified water column (Figure 6, right panel). Consequently, this may lead to lower primary production compared with areas directly affected by marine‐terminating glaciers (Hopwood et al., 2020; Meire et al., 2017).

Besides upwelling, we found the prevailing current system and the intrusion of AW to influence the distribution of freshwater and therefore biogeochemical cycles. The current system determines how the meltwater plume propagates and what areas are affected by glacial runoff. The influence of freshwater originating from marine‐terminating glaciers decreases along the fjord axis as freshwater progressively mixes with fjord waters. The intrusion of warmer AW in subsurface waters acts as a boundary layer and restricts advection of deeper waters in the outer fjord (Figure 6, left panel).

At the sediment‐water interface, we suggest benthic fluxes attributed to the dissolution of labile particles and biotic/abiotic redox reactions in the sediment are an important source of silicate, dFe, dMn, dCu, and dZn to the water column (Figure 6, both panels). Previous studies showed that the flux of dFe depends on the interplay between sedimentation rates and organic carbon availability, with the highest fluxes in the mid‐fjord region (Herbert et al., 2021). We hypothesize that benthic fluxes of silicate, dFe, dMn, dCu, and dZn could be higher close to land‐terminating glaciers, since more reactive particulate trace element species are generated by proglacial and riverine processes. This might drive benthic cycling and could lead to increased remobilization from the sediment (Herbert et al., 2020).

Beneficial for future research would be a higher temporal and spatial scale of sampling. Our study was limited to a certain number of stations sampled during a relatively short period of time in summer. The seasonal mean temperature during summer 2020 was very high, resulting in large meltwater fluxes. It would be interesting to compare the results with a year of contrasting conditions, ideally over a longer period of time, to also capture seasonal changes.

### 4.2 Future of Fjord Systems

Considering the Kongsfjorden system to be an early warning indicator of future changes (Bischof et al., 2019), we can hypothesize possible climate change‐induced transformations and extrapolate a pan‐Arctic perspective. Our results suggest that the type of glacier present in a fjord affects nutrient availability and therefore primary production. Marine‐terminating glaciers of Kongsfjorden are retreating and are expected to become land‐terminating at some point in the future (Torsvik et al., 2019). The transition to a fjord with only land‐terminating glaciers will likely enhance stratification during summer months, especially in the inner parts of the fjord, as mixing will become less turbulent at the glacier fronts. The combination of increasing stratification and decreasing upwelling will likely limit the supply of nutrients to surface waters, altering primary production (Hopwood et al., 2020; Meire et al., 2017). Hence, persistent glacier retreat of Arctic fjord systems might lead to overall less productive ecosystems in the future. In terms of trace element distributions, we hypothesize that the overall input of dissolved elements will decrease since proglacial rivers exhibit lower trace element concentrations than discharge from marine‐terminating glaciers. In contrast, benthic fluxes of trace elements might increase as more reactive trace metal phases are transported by proglacial rivers that could potentially drive increased benthic cycling (Herbert et al., 2020). Other studies have shown that benthic iron fluxes might be sensitive to future glacial retreat with fjord sediments functioning less as a source of bioavailable iron, which might further impact primary production in fjord systems (Herbert et al., 2021; Laufer‐Meiser et al., 2021; Wehrmann et al., 2014).

The transition to a more proglacial discharge dominated system could provide less positive feedback to OA (Fransson et al., 2015), as higher buffering capacities in proglacial river water could mitigate freshwater dilution by discharge from marine‐terminating glaciers. An overall decrease in photosynthetic CO_2_ uptake has consequences for the carbonate system, which could lead to increasing acidification of fjord waters (Hopwood et al., 2020). Overall, we expect a transition from marine‐ to land‐terminating glaciers to negatively affect the productivity of Arctic fjord systems, with larger implications for the biogeochemical composition of the water column, which will likely impact biotic and abiotic carbon uptake in the future.

## Conflict of Interest

The authors declare no conflicts of interest relevant to this study.

## Supporting information

Supporting Information S1

## Data Availability

The data set for this article can be found online at https://doi.org/10.5281/zenodo.11473603 (Schmidt et al., 2024).

## Appendix A Statistical Methods

Principal component analysis (PCA) is a statistical technique that converts a set of intercorrelated variables ($Z_{ik}$) into linearly uncorrelated principal components (PCs). The objective is to create PCs that are linear combinations of the original input variables, where the variance between the PCs and the original variables is maximized. The first step in this analysis is transforming the concentration data of the parameters $C_{ik}$ into the standardized form $Z_{ik}$.

(A1)
$$Z_{ik}=\frac{\left(C_{ik}-\bar{C_{i}}\right)}{\sigma_{i}}$$

In this equation, i=1,2,…n is the total number of parameters, k=1,2,…m is the total number of observations, $\bar{C_{i}}$ is the mean concentration for each parameter over all observations, and $\sigma_{i}$ is the standard deviation of the concentration of each parameter.

In order to maximize the variance, the correlation matrix between the variables [X] is decomposed by extracting eigenvectors [W] and eigenvalues *λ* that satisfy the following equations:

(A2)
$${\left[X\right]}_{ixi}={\left[Z\right]}_{ixk}{\left[Z\right]}_{ixk}^{\prime }$$

(A3)
$${\left[X\right]}_{ixi}{\left[W\right]}_{jxi}={\left[\lambda \right]}_{j}{\left[X\right]}_{ixi}$$

The correlation of each variable with each PC is expressed by PC loadings [B], which are calculated by multiplying eigenvectors [W] with the square root of eigenvalues *λ*.

(A4)
$${\left[B\right]}_{jxi}={\left[W\right]}_{jxi}\sqrt{\lambda_{j}}$$

The original data matrix [Z] can be expressed as a linear combination of PC scores [P] with PC loadings [B] as their coefficients.

(A5)
$${\left[Z\right]}_{ixk}={\left[P\right]}_{jxk}{\left[B\right]}_{jxi}$$

The rotation of PCs can simplify the interpretation of the components. Therefore, varimax rotation was used as a criterion to calculate rotated PC loadings ${\left[B\right]}^{*}$. Rotated PC scores ${\left[P\right]}^{*}$ can be computed using the rotated ${\left[B\right]}^{*}$ matrix.

(A6)
$${\left[P\right]}_{jxk}^{*}={\left[B\right]}_{jxi}^{*}{\left[Z\right]}_{ixk}$$

The rotated PC scores ${\left[P\right]}^{*}$ are correlated with respective impact sources j affecting the concentration of a parameter with j=1,2,…p. A higher component score $P_{jk}^{*}$ implies a higher influence by the impact source j during observation k. However, PC scores are not proportional to impact sources since they are computed using standardized concentrations $Z_{ik}$. This prevents a direct apportionment of changes in concentrations to impact source components. Previous work by Thurston and Spengler (1985) uses “absolute zero” PC scores $P_{0,p}^{*}$ to convert standardized PC scores into the non‐standardized Absolute PC Scores ${\text{APCS}}_{jk}^{*}$. Absolute zero PC scores for each component p are derived from *Z*‐scores for absolute zero concentrations ${\left(Z_{0}\right)}_{i}$.

(A7)
$${\left[P_{0,p}^{*}\right]}_{jxk}={\left[B_{0}\right]}_{jxi}^{*}{\left[Z\right]}_{ixk}$$

where

(A8)
$${\left[B_{0}\right]}_{jxi}^{*}={\left[B\right]}_{jxi}^{*}{\left(Z_{0}\right)}_{i}$$

and

(A9)
$${\left(Z_{0}\right)}_{i}=-\frac{{\bar{C}}_{i}}{\sigma_{i}}$$

The APCS scores for each component are estimated as follows:

(A10)
$${\left[\text{APCS}\right]}_{jxk}^{*}={\left[P\right]}_{jxk}^{*}-{\left[P_{0,p}^{*}\right]}_{jxk}$$

The observed concentration of the parameter $C_{ik}$ can be expressed as a linear combination of the different processes, expressed in the following equation:

(A11)
$$C_{ik}=\alpha_{0}+\sum_{j=1}^{p}\alpha_{ij}\;{APCS}_{jk}^{*}$$

where $\alpha_{0}$ is the mean concentration over all observations that is not influenced by any processes and $\alpha_{ij}\;{\text{APCS}}_{jk}^{*}$ is the contribution made by the process j on observation k from $\alpha_{0}$.The contribution of each process can be quantified by multiple linear regression (MLR) of the observed concentration data on the APCS scores and solving for the coefficients $\alpha_{ij}$ and the constant $\alpha_{0}$. This allows calculation of the percentage of overall change in distribution made by all processes $R_{\text{tot}}$ and the percentage of each process on the overall change in distribution $R_{P}$.

(A12)
$$R_{\text{tot}}\left[\%\right]=\frac{\sum_{j=1}^{p}\left|\bar{\alpha_{ij}\;{\text{APCS}}_{jk}^{*}}\right|}{\sum_{j=1}^{p}\left|\bar{\alpha_{ij}\;{\text{APCS}}_{jk}^{*}}\right|+\alpha_{0}}\times 100$$

(A13)
$$R_{P}\left[\%\right]=\frac{\bar{\left|\alpha_{ij}\;{\text{APCS}}_{jk}^{*}\right|}}{\sum_{j=1}^{p}\left|\bar{\alpha_{ij}\;{\text{APCS}}_{jk}^{*}}\right|+\alpha_{0}}\times 100$$

The ratio between $R_{P}$ and $R_{\text{tot}}$ shows the importance of each individual process compared to the other processes.

(A14)
$$R_{S}\left[\%\right]=\frac{R_{P}}{R_{\text{tot}}}=\frac{\bar{\left|\alpha_{ij}\;{\text{APCS}}_{jk}^{*}\right|}}{\sum_{j=1}^{p}\left|\bar{\alpha_{ij}\;{\text{APCS}}_{jk}^{*}}\right|}\times 100$$

## Appendix B Calculation of the Freshwater Content

The freshwater content of the fjord is calculated following the methodology described in the literature (Beszczynska‐Möller et al., 1997; Promińska et al., 2017). The freshwater content (FWC) of each station was calculated by integrating the measured salinity relative to the reference salinity (34.86) over the water column:

(B1)
$$\text{FWC}\left[m\right]=\int_{z}^{0}\frac{S_{\text{ref}}-S}{S_{\text{ref}}}dz$$

The specific freshwater content (FWC_sp_) of each station is calculated by integrating the measured salinity relative to the reference salinity (34.86) over the water column and averaging by depth [m]:

(B2)
$${\text{FWC}}_{\text{sp}}\left[\%\right]=\left(\frac{1}{z}\;\int_{z}^{0}\frac{S_{\text{ref}}-S}{S_{\text{ref}}}dz\;\right)\times 100$$

where FWC_sp_ is the proportion of freshwater of each station, *S*_ref_ is the reference salinity, *S* is the measured salinity from CTD data and *z* is the depth [m]. The reference salinity was obtained by calculating the median of the values in the deeper layer (100–245 m depth) of the fjord mouth (station T).
