# Supplementary material for: The Contrasting Role of Marine‐ and Land‐Terminating Glaciers on Biogeochemical Cycles in Kongsfjorden, Svalbard
Source: Global Biogeochem Cycles. 2025 Jan 6;39(1):e2023GB008087. doi: 10.1029/2023GB008087 (PMC11702319; doi:10.1029/2023GB008087)
Supplement: Supplementary file 1 — Supporting Information S1 [file GBC-39-0-s001.docx]

*Global Biogeochemical Cycles*

Supporting Information for

**The Contrasting Role of Marine- and Land-terminating Glaciers on Biogeochemical Cycles in Kongsfjorden, Svalbard**

C. E. Schmidt^1,2^, D. Pröfrock^3^, G. Steinhoefel^4^, T. Stichel^4^, C. Mears^1^, L. M. Wehrmann^5^, and H. Thomas^1,2^

^1^Helmholtz-Zentrum Hereon, Institute of Carbon Cycles, D-21502, Geesthacht

^2^Carl von Ossietzky University Oldenburg, Institute for Chemistry and Biology of the Marine Environment, D-26129, Oldenburg, Germany

^3^Helmholtz-Zentrum, Institute of Coastal Environmental Chemistry, D-21502, Geesthacht, Germany

^4^Alfred Wegener Institute, Helmholtz Center for Polar and Marine Research, D-27570, Bremerhaven, Germany

^5^Stony Brook University, School of Marine and Atmospheric Sciences, NY 11794, Stony Brook, USA

**Contents of this file**

Figures S1 to S3

Tables S1 to S9

**Additional Supporting Information (Files uploaded separately)**


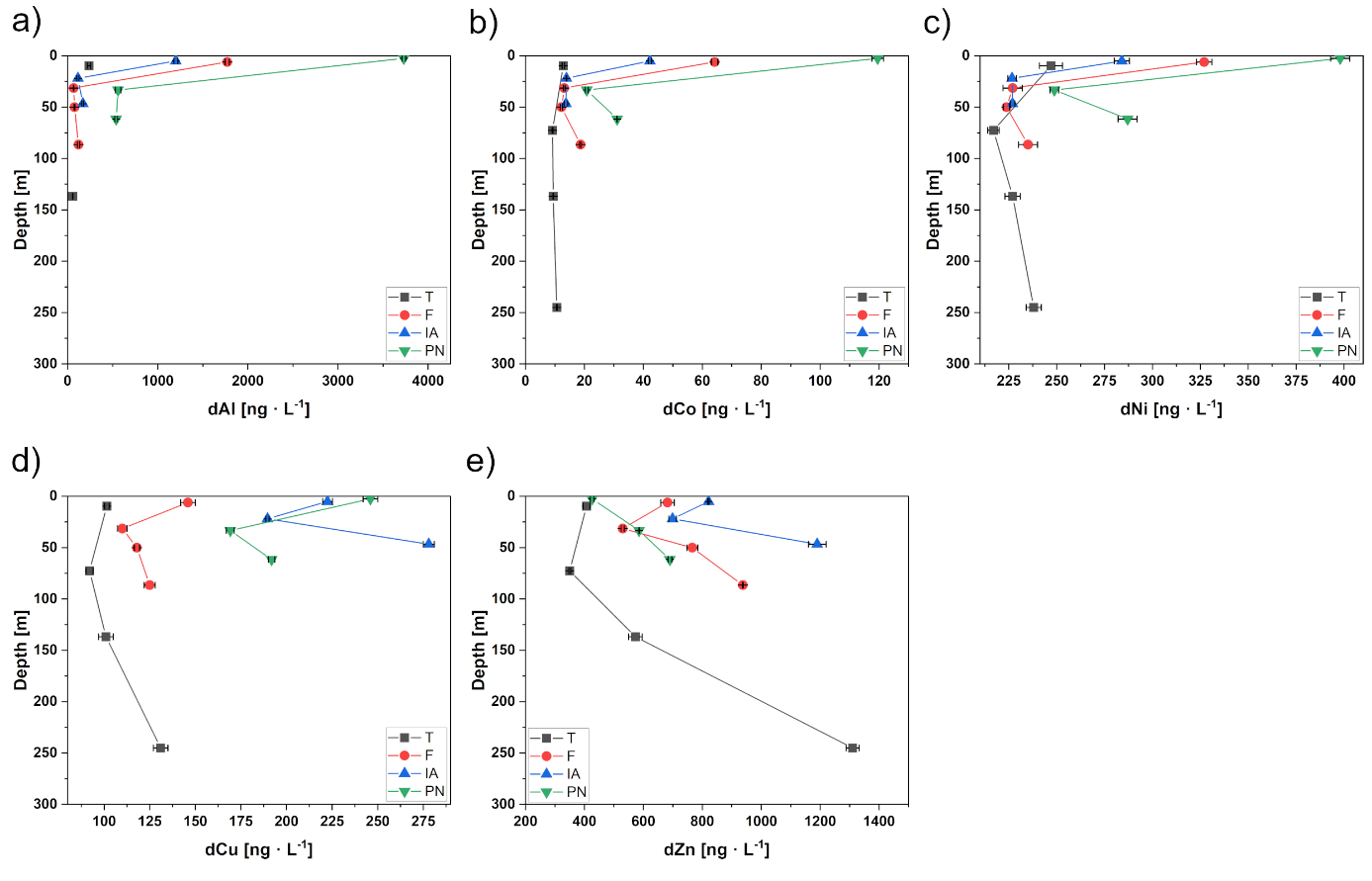


**Figure S1.** Additional depth profiles of measured elements in Kongsfjorden for stations T (black), F (red), IA (blue) and PN (green). Shown are dissolved elements: a) Aluminum (dAl), b) Cobalt (dCo), c) Nickel (dNi), d) Copper (dCu) and e) Zinc (dZn). Values below LOQ are not shown. Error bars correspond to U (k = 2).


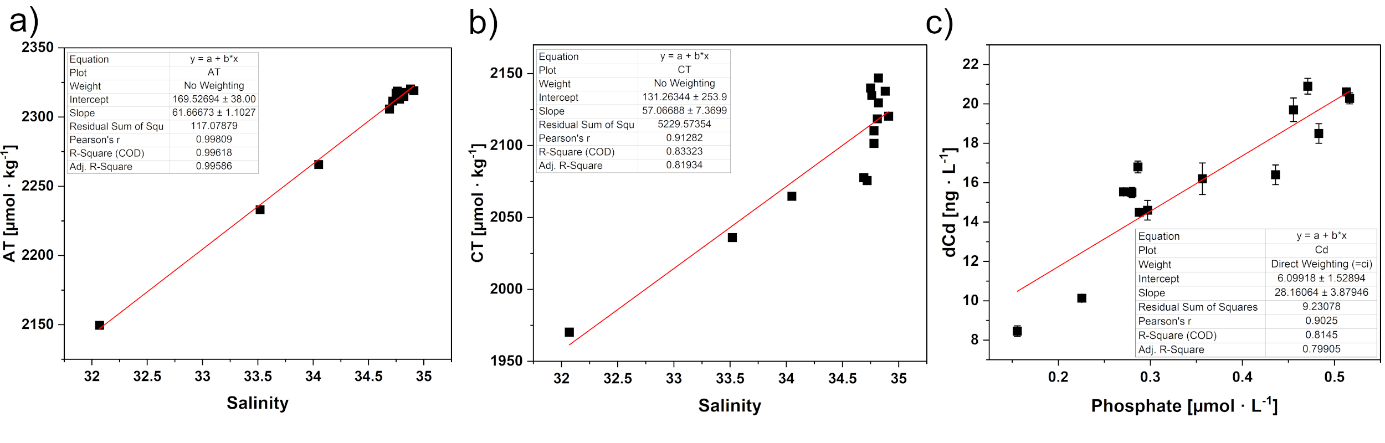
**Figure S2.** Linear regression of a) AT vs Salinity, b) CT vs Salinity and c) dCd vs Phosphate.


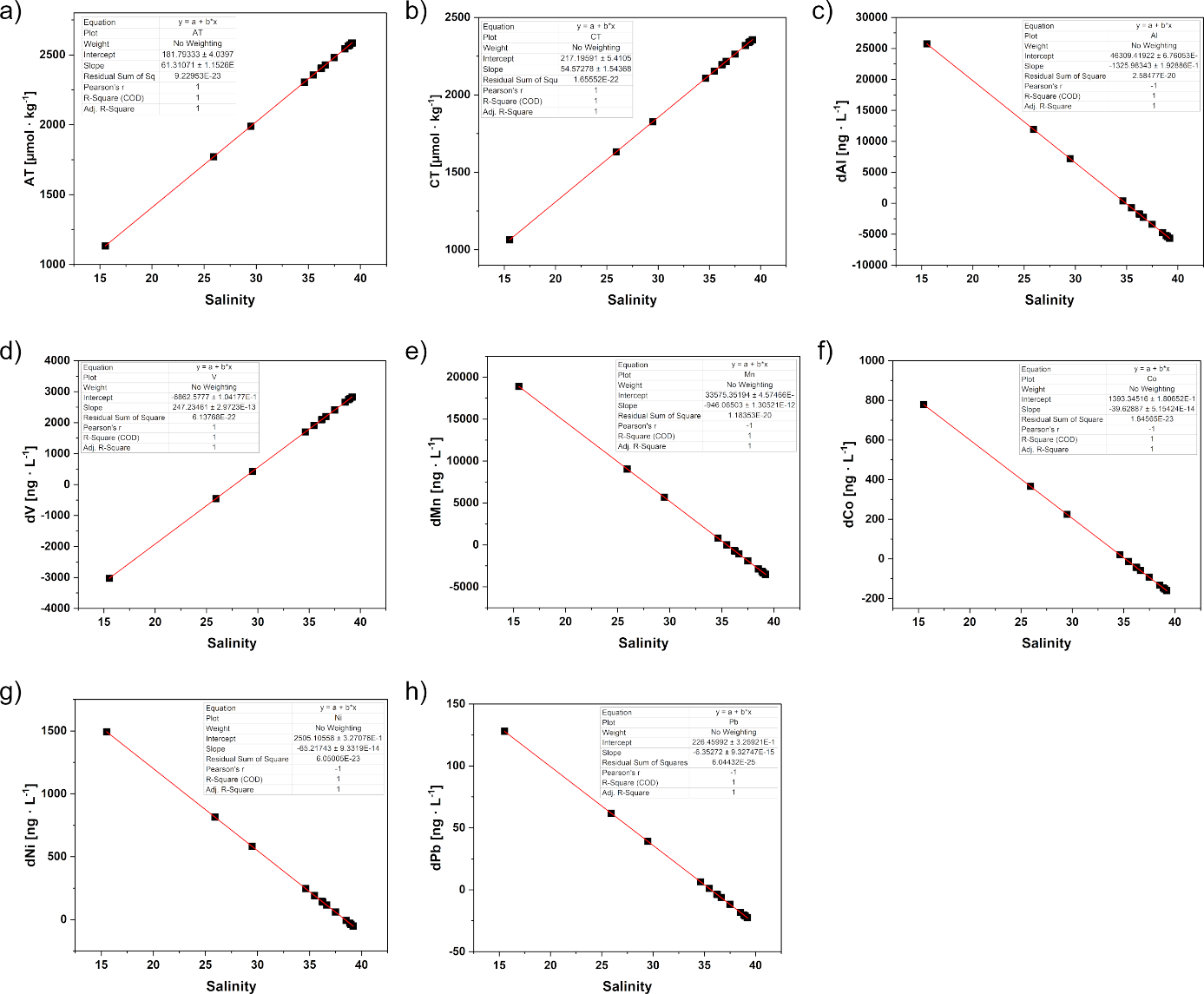


Figure S3. Linear regression of de-normalized partial values against salinity for robust endmember relationships.

Table S1. Summary of the sampling locations in the fjord (T, F, IA and PN) and outflow of proglacial catchments (ML, AL and BR) during July 2020.

| Station | Sample ID | Sampling Date | Latitude [°N] | Longitude [°E] | Total depth [m] | Water depth [m] |
| --- | --- | --- | --- | --- | --- | --- |
| T | T_10  T_73  T_137  T_245 | 03 July 2020 | 78.96549 | 11.87576 | 342 | 9.7  72.6  136.8  245.1 |
| F | F_6  F_31  F_50  F_87 | 01 July 2020 | 78.91808 | 12.24499 | 112 | 6.2  31.4  50.1  86.5 |
| IA | IA_5  IA_22  IA_47 | 06 July 2020 | 78.89641 | 12.33456 | 54 | 5.1  22.0  46.7 |
| PN | PN_3  PN_33  PN_62 | 06 July 2020 | 78.89416 | 12.49862 | 76 | 2.5  33.3  61.6 |
| ML | ML | 02 July 2020 | 78.91106 | 12.10145 | ‒ | Surface |
| AL | AL | 05 July 2020 | 78.903384 | 12.15234 | ‒ | Surface |
| BR | BR | 09 July 2020 | 78.93506 | 11.86327 | ‒ | Surface |

Table S2. Instrument settings of ICP-MS system.

| ICP-MS system | Agilent 7900 Single Quadrupole ICP-MS (Agilent Technologies) |
| --- | --- |
| Sample preconcentration | seaFAST SP2 (Elemental Scientific) |
| Interface cone | Ni |
| Lense | x-lense |
| RF power | 1550 W |
| Nebulizer pump | 0.10 rps |
| Carrier gas flow | 1.00 L · min^-1^ |
| Makeup gas flow | 0.11 L · min^-1^ |
| Cell gas 1: He | 4.0 mL · min^-1^ |
| Cell gas 2: H_2_ | 0.5 mL · min^-1^ |
| Purity cell gases | 5.0 |
| Integration time | 50 ms |
| Measured isotopes | ^27^Al, ^51^V, ^55^Mn, ^56^Fe, ^59^Co, ^60^Ni, ^65^Cu, ^66^Zn, ^111^Cd, ^208^Pb |
| Extract 1 | 0.0 V |
| Extract 2 | -185.0 V |
| Omega Bias | -85 V |
| Omega Lens | 10.0 V |
| Q1 Entrance | -70 V |
| Q1 Exit | -80 V |
| Deflect | 5.0 V |
| Plate Bias | -65 V |
| OctP Bias | -18.0 V |
| OctP RF | 200 V |
| Energy Discrimination | 5.0 V |

Table S3. Validation summary of multi-element analysis.

| Element | Fjord water | | River water | | Recovery [%] | | | |
| --- | --- | --- | --- | --- | --- | --- | --- | --- |
|  | LOD  [ng · L^-1^] | LOQ  [ng · L^-1^] | LOD  [ng · L^-1^] | LOQ  [ng · L^-1^] | CASS-6 | NASS-7 | SLRS-6 | KBA-QC |
| dAl | 30 | 50 | 180 | 500 | - | - | 117 | 114 |
| dV | 5 | 15 | 0.4 | 1 | 89 | 115 | 106 | 128 |
| dFe | 22 | 29 | 9 | 31 | 108 | 109 | 107 | 102 |
| dMn | 0.8 | 1.9 | 250 | 790 | 106 | 113 | 106 | 105 |
| dCo | 0.03 | 0.07 | 0.3 | 1 | 109 | 110 | - | 106 |
| dNi | 0.3 | 0.8 | 0.8 | 2.3 | 112 | 110 | 103 | 107 |
| dCu | 0.4 | 0.6 | 2.4 | 6 | 112 | 107 | 109 | 107 |
| dZn | 4 | 8 | 6 | 15 | 112 | 102 | 106 | 102 |
| dCd | 0.017 | 0.05 | 0.04 | 0.13 | 113 | 102 | 128 | 103 |
| dPb | 0.16 | 0.28 | 0.7 | 1.9 | 114 | 93 | 99 | 100 |
| Nutrient | LOD [µg · L^-1^] | | | | Lot: BZ | | Lot: CL | |
| Nitrate | 0.03 | | | | 101 | | 91 | |
| Nitrite | 0.01 | | | | 108 | | 80 | |
| Silicate | 1.2 | | | | 96 | | 96 | |
| Phosphate | 0.01 | | | | 99 | | 95 | |

Table S4 Results of the Shapiro-Wilk test of normality with a critical value $\boldsymbol{W}_{\boldsymbol{\alpha}}$ = 0.877 (α = 0.05, n = 14).

| Parameter | *W* | $H_{0}$ |
| --- | --- | --- |
| Salinity | 0.586 | rejected |
| Temperature | 0.896 | accepted |
| Nitrate | 0.896 | accepted |
| Nitrite | 0.932 | accepted |
| Silicate | 0.942 | accepted |
| Phosphate | 0.915 | accepted |
| AT | 0.589 | rejected |
| CT | 0.857 | accepted |
| dAl | 0.630 | rejected |
| dV | 0.816 | rejected |
| dFe | 0.934 | accepted |
| dMn | 0.762 | rejected |
| dCo | 0.648 | rejected |
| dNi | 0.746 | rejected |
| dCu | 0.909 | accepted |
| dZn | 0.924 | accepted |
| dCd | 0.918 | accepted |
| dPb | 0.746 | rejected |

*Note.* The null-hypothesis ${(H}_{0})$ of this test is that the population is normally distributed. Thus, if the test value $W$is less than the critical value $W_{\alpha}$ (at α = 0.05), then the null hypothesis is rejected and there is evidence that the data tested are not normally distributed. The test value W of temperature, nitrate, nitrite, silicate, phosphate, CT, dFe, dCu, dZn, dCd is larger than the critical value W_α and thus variables are normally distributed. For salinity, AT, dAl, dV, dMn, dCo, dNi, dPb the null hypothesis is rejected and thus variables are not normally distributed.

Table S5 Results of the $\boldsymbol{X}^{\mathbf{2}}$ independence test for each pair of components with a critical value $\boldsymbol{X}_{\boldsymbol{\alpha}}^{\mathbf{2}}$ = 7.815 (α = 0.5, n = 36).

|  | PC1 / PC 2 | PC1 / PC 3 | PC 2 / PC 3 |
| --- | --- | --- | --- |
| $X^{2}$ | 8.154 | 9.921 | 7.911 |
| $H_{0}$ | accepted | accepted | accepted |

*Note.* The null-hypothesis $(H_{0})$ of this test is that the components are independent from each other. Thus, if the test value $X^{2}$ is less than the critical value $X_{\alpha}^{2}$ (at α = 0.5), then the null hypothesis is rejected and there is evidence that the components tested are not independent. The $X^{2}$ values of PC pairs are larger than the critical value $X_{\alpha}^{2}$, which implies the independence of PCs, thus each PC provides unique information for each dimension.

Table S6. Water masses and their characteristic temperature-salinity ranges of Kongsfjorden based on Cottier et al. (2005).

| Water masses in Kongsfjorden | Temperature [°C] | Salinity [PSU] |
| --- | --- | --- |
| Local Water LW | 0.5 < T < 1.0 | 34.30 < S < 34.85 |
| Surface Water SW | T > 1.0 | S < 34.00 |
| Transformed Atlantic Water TAW | 1.0 < T < 3.0 | S > 34.65 |
| Intermediate Water IW | T > 1.0 | 34.00 < S < 34.65 |
| Atlantic Water AW | T > 3.0 | S > 34.65 |

Table S7. Freshwater content (FWC) and specific freshwater content of each station (FWC*_sp_*). Calculation according to Beszczynska-Möller et al. (1997) and Promińska et al. (2017).

| Station | FWC [m] | FWC_sp_ [%] |
| --- | --- | --- |
| T | 0.37 | 0.15 |
| F | 0.68 | 0.78 |
| IA | 0.43 | 0.96 |
| PN | 0.90 | 1.5 |

Table S8. Concentrations of nutrients and carbon parameters given as minimum and maximum over all fjord stations and concentrations of proglacial rivers from Midre Lovénbreen (ML), Austre Lovénbreen (AL) and Bayelva River (BR) catchments. The station ID in brackets corresponds to the station name and sampling depth connected by an underscore. Uncertainties are reported with a coverage factor k = 2.

| Ω |  | 1.86  [T_245] | 2.47  [T_10] | - | | - | - |  |
| --- | --- | --- | --- | --- | --- | --- | --- | --- |
| pCO_2_ | [µatm] | 222  [T_10] | 286  [T_137] | - | | - | - |  |
| pH |  | 8.16  [T_245] | 8.26  [T_10] | 8.12 | | 8.43 | 8.01 |  |
| CT | [µmol · kg^-1^] | 1970  [PN_3] | 2147  [T_245] | 1031 | | 953 | 966 |  |
| AT |  | 2150  [PN_3] | 2320  [T_137] | 1267 | | 1261 | 1305 |  |
| Phos-phate | [µmol · L^-1^] | 0.16 ± 0.005  [T_10] | 0.52 ± 0.003  [F_87] | 0.16 ± 0.01 | | 0.06 ± 0.02 | 0.04 ± 0.01 |  |
| Silicate |  | 0.58 ± 0.019  [T_10] | 1.69 ± 0.022  [F_87] | 6.67 ± 0.18 | | 7.44 ± 0.35 | 4.76 ± 0.18 |  |
| Nitrite |  | < 0.01 (LOD)  [IA_22; T_10] | 0.11 ± 0.001  [T_245] | 0.40 ± 0.008 | | 0.06 ± 0.002 | 0.02 ± 0.001 |  |
| Nitrate |  | < 0.02 (LOD)  [IA_22; IA_47; T_10) | 3.81 ± 0.028  [T_245] | 1.71 ± 0.026 | | 0.77 ± 0.017 | 1.29 ± 0.002 |  |
| Temper-ature | [°C] | 0.75  [T_245] | 3.98  [IA_22] | 2.9 | | 2.8 | 5.0 |  |
| Salinity |  | 32.07  [PN_3] | 34.91  [T_73] | 0.06 | | 0.05 | 0.05 |  |
| Parameter | Units | Min | Max | ML | | AL | BR |  |
|  |  | Kongs-fjorden | | | Proglacial Rivers | | | |

Table S9. Concentrations of dissolved elements given as minimum and maximum over all fjord stations and concentrations of proglacial rivers from Midre Lovénbreen (ML), Austre Lovénbreen (AL) and Bayelva River (BR) catchments. The station ID in brackets corresponds to the station name and sampling depth connected by an underscore. Uncertainties are reported with a coverage factor k = 2.

| dPb | [ng · L^-1^] | 2.43 ± 0.28  [T_73] | 17.8 ± 0.4  [F_6] | 4.1 ± 0.3 | 98 ± 7 | <1.9 (LOQ) |
| --- | --- | --- | --- | --- | --- | --- |
| dCd |  | 8.46 ± 0.27  [T_10] | 20.9 ± 0.4  [PN_62] | 1.9 ± 0.28 | 5.04 ± 0.23 | 4.5 ± 0.4 |
| dZn |  | 350 ± 3  [T_73] | 1310 ± 22  [T_245] | 49.2 ± 1.3 | 76.4 ± 2.0 | 53.3 ± 1.3 |
| dCu |  | 92 ± 1.9  [T_73] | 278 ± 3  [IA_47] | 60.8 ± 2.1 | 111 ± 6 | 87.7 ± 1.6 |
| dNi |  | 217 ± 3  [T_73] | 398 ± 5  [PN_3] | 140 ± 6 | 202 ± 12 | 36.2 ± 0.4 |
| dCo |  | 9.17 ± 0.16  [T_73] | 119.5 ± 2.0  [PN_3] | 50.4 ± 1.0 | 23.8 ± 0.9 | 15.37 ± 0.24 |
| dMn |  | 330 ± 6  [T_137] | 3180 ± 60  [PN_3] | 22,000 ± 600 | 6850 ± 250 | 9710 ± 210 |
| dFe |  | 101.7 ± 2.4  [T_137] | 205 ± 9  [IA_47] | 3360 ± 110 | 7400 ± 300 | 348 ± 3 |
| dV |  | 1170 ± 40  [PN_3] | 1840 ± 90  [T_73] | 54.0 ± 1.6 | 30.1 ± 1.2 | 29.8 ± 0.5 |
| dAl |  | < 50 (LOQ)  [T_73; T_245] | 3730 ± 20  [PN_3] | 24,300 ± 500 | 45,200 ± 900 | 9020 ± 240 |
| Parameter | Units | Min | Max | ML | AL | BR |
|  |  | Kongs-fjorden | | Proglacial Rivers | | |
